# *Rheum tanguticum* Alleviates Cognitive Impairment in APP/PS1 Mice by Regulating Drug-Responsive Bacteria and Their Corresponding Microbial Metabolites

**DOI:** 10.3389/fphar.2021.766120

**Published:** 2021-12-15

**Authors:** Demin Gao, Huizhen Zhao, Zhihui Yin, Chen Han, Ying Wang, Gan Luo, Xiaoyan Gao

**Affiliations:** School of Chinese Materia Medica, Beijing University of Chinese Medicine, Beijing, China

**Keywords:** Alzheimer’s disease, rhubarb, gut microbiota, drug-responsive bacteria, *o*-tyrosine

## Abstract

Drugs targeting intestinal bacteria have shown great efficacy for alleviating symptoms of Alzheimer’s disease (AD), and microbial metabolites are important messengers. Our previous work indicated that *Rheum tanguticum* effectively improved cognitive function and reshaped the gut microbial homeostasis in AD rats. However, its therapeutic mechanisms remain unclear. Herein, this study aimed to elaborate the mechanisms of rhubarb for the treatment of AD by identifying effective metabolites associated with rhubarb-responsive bacteria. The results found that rhubarb reduced hippocampal inflammation and neuronal damage in APP/PS1 transgenic (Tg) mice. 16S rRNA sequencing and metabolomic analysis revealed that gut microbiota and their metabolism in Tg mice were disturbed in an age-dependent manner. Rhubarb-responsive bacteria were further identified by real-time polymerase chain reaction (RT-PCR) sequencing. Four different metabolites reversed by rhubarb were found in the position of the important nodes on rhubarb-responsive bacteria and their corresponding metabolites combined with pathological indicators co-network. Furthermore, *in vitro* experiments demonstrated *o*-tyrosine not only inhibited the viabilities of primary neurons as well as BV-2 cells, but also increased the levels of intracellular reactive oxygen species and nitric oxide. In the end, the results suggest that rhubarb ameliorates cognitive impairment in Tg mice through decreasing the abundance of *o*-tyrosine in the gut owing to the regulation of rhubarb-responsive bacteria. Our study provides a promising strategy for elaborating therapeutic mechanisms of bacteria-targeted drugs for AD.

## Introduction

Alzheimer’s disease (AD) is a progressive neurodegenerative disease with high incidence, disability and death rates (Sierksma et al., 2020). Clinically, AD patients are characterized by age-dependent memory loss, cognitive dysfunction and behavior abnormality (Jack et al., 2018). It is widely believed that the pathological changes in the brains of AD patients belong to the accumulated amyloid-β (Aβ) induced oxidative stress and inflammatory responses in brain, causing oxidative damage and release of pro-inflammatory mediators, finally neuronal apoptosis (Stepanichev et al., 2004; Pistollato et al., 2016; Wu Y. et al., 2019). Emerging evidence has shown that gut microbial dysbiosis may mediate the pathogenesis of AD (Minter et al., 2016; Liu P. et al., 2019; Wang et al., 2019). Therefore, remodeling gut microbial homeostasis may represent a more effective therapeutic strategy for the treatment of AD.

Traditional Chinese medicine (TCM) has aroused increasing attention on account of its therapeutic effects in the prevention and treatment of AD (Xu et al., 2017). Among them, rhubarb exhibits excellent therapeutic effect for AD by “tonify the body by removing stasis” and “relaxing bowels and puzzle” according to “brain collateral damage due to toxin” raised by theoretical system of TCM (Li et al., 2019). Our previous studies have suggested that *Rheum tanguticum* effectively improves cognitive function and reshapes the gut microbial homeostasis in AD animal models (Zhao et al., 2019). However, its therapeutic mechanisms remain unclear. Herein, considering the fact that a relatively low content of chemical ingredients in rhubarb permeated into the brain (Sun et al., 2012; Dong et al., 2016), we hypothesized that therapeutic effects of rhubarb may attribute to remodeling gut microbiota. Thus, it is of great significance to the elaborating therapeutic mechanism of rhubarb for AD from the view of gut microbiota (Mancuso and Santangelo, 2018).

Among the researches involved in interaction between gut and brain, a growing body of evidence suggests that microbial metabolites are important messengers of intestinal bacteria responsible for regulation of physiological state in brain, although the mechanisms of gut-brain transmission have so far remained elusive (Nicholson et al., 2012; Rothhammer et al., 2018). It has been found that harmful microbial metabolites, such as lipopolysaccharides (LPS) and Trimethylamine-*N*-oxide (Vogt et al., 2018), are easy to enter the brain and destroy its homeostasis, because intestinal leakage and permeability of BBB are significantly increased in AD patients (Kim et al., 2020). Accordingly, the regulation of gut microbiota-derived microbial metabolites is beneficial to alleviating symptoms of AD. Therefore, accurate identification of microbial metabolites associated with drug-responsive bacteria is crucial to elucidate the mechanisms of bacteria-targeted drugs for the treatment of AD.

In terms of the progression in a disease as well as drug intervention, metabolomics is a powerful tool for comprehensive discovery of the disturbed metabolites in a biological system (Matysik et al., 2016; Shaffer et al., 2017; Luan et al., 2019). However, it fails to distinguish the metabolites derived from drug-responsive bacteria. Because of the lack of direct identification methods, the alternative methods are diverse bacteria-based correlation analysis and co-network analysis combined with pathophysiological indicators (Feng et al., 2019; Zheng et al., 2020). Over the last decade, microbiome research based on amplicon sequencing, such as 16S rRNA, offers the global relative abundance of bacteria in different taxonomies (Gohl et al., 2016). However, comprehensive yet redundant data hinder accurate quantification of the drug-responsive bacteria. Given that relative abundance of bacteria could not reflect actual content of gut microbiota by 16S rRNA sequencing (Eisenstein, 2018; Tkacz et al., 2018), meanwhile, uncertainty exists in peak area of the fragment ions and their real content in biological samples by metabolomics analysis (Luan et al., 2019), there would be many false positives in identified microbial metabolites by 16S rRNA sequencing-based co-network analysis (Peisl et al., 2018). Thus, it always gives obscure explanation of the therapeutic mechanisms of bacteria-targeted drugs. Therefore, it is urgent to improve the accuracy of identification for microbial metabolites associated with drug-responsive bacteria.

In recent years, real-time polymerase chain reaction (RT-PCR) has been adopted for quantifying microbiota significantly disturbed between the health individuals and patients (Tettamanti Boshier et al., 2020). Based on the filtered specific intestinal bacteria in disease by 16S rRNA, RT-PCR as a complementary technology can obtain more precise drug-responsive bacteria by semi-quantitative comparative analysis between the treatment group and the disease group (Neyrinck et al., 2017; Xu et al., 2021). To some extent, the above-mentioned method can eliminate false positive bacteria and contribute to unravel therapeutic mechanisms of bacteria-targeted drugs in a more accurate way. Hence, a co-network analysis proposed here, which consists of RT-PCR and different metabolites combined with measurable pathological indicators, can immensely improve the accuracy of identification for microbial metabolites associated with drug-responsive bacteria.

Based on the proposed strategy, the present study aimed to elaborate the mechanism of rhubarb for AD by identifying effective metabolites associated with rhubarb-responsive bacteria. First of all, the influence of long-term rhubarb treatment on the pathological indicator-related microbiota and corresponding metabolites was evaluated. And then, gut microbiota and metabolomic profiling based on a time series analysis were performed to filter the disturbed microbiota and metabolites with the progression of AD. Moreover, the rhubarb-responsive bacteria and their corresponding metabolites were discovered by the co-network analysis based on RT-PCR and different metabolites combined with pathological indicators. Finally, the effects of key metabolite on primary neurons and BV-2 cells were evaluated *in vitro* to validate the bidirectional cross-talk between the gut and brain. Together, a more accurate co-network analysis employed in our study demonstrates that rhubarb ameliorates cognitive impairment by influencing effective metabolites in the gut through the regulation of rhubarb-responsive bacteria.

## Materials and Methods

### Animals

Eight-month-old male APP/PS1 Tg mice and littermate wild type (WT) mice were obtained from the Nanjing Biochemical Research Institute of Nanjing University and housed in environmentally controlled conditions (room temperature at 22 ± 1°С, 12-h light/dark cycle) with access to standard food and water ad libitum. All experiments were approved and conducted in accordance with the guidelines of the Care and Use of Laboratory Animals approved by the Ethics Committee for Animal Care and Treatment at Beijing University of Chinese Medicine (BUCM-4-2018080101-3007).

### Preparation of Drug Solutions

#### Preparation of Rhubarb Decoction

Small pieces of rhubarb (*Rheum tanguticum*; 171202 ZISUN MEDICINE HEALTH CO.LTD. GuangZhou, China) herbal medicine (∼30 g) were weighed and soaked in 300 ml distilled water for 1 h and decocted twice in boiling water for 30 min on each occasion. The combined products from this water decoction were filtered and dried in a 60°C water bath, then topped up to 50 ml with ultrapure water to yield a 0.6 g mL^−1^ solution (Zhao et al., 2019).

#### Preparation of Donepezil Hydrochloride Solution

Donepezil hydrochloride solution was prepared by crushing one tablet containing 5 mg donepezil hydrochloride and dissolving it in 100 ml of normal saline by ultrasonication for 30 min to obtain a 0.05 mg ml^−1^ solution.

### Experimental Design

All the 8-month-old Tg mice were randomly divided into three groups (*n* = 9 per group): Tg model group, rhubarb administration group (TgR), and positive drug group (TgP). Littermate wild type mice (*n* = 9) were used as the wild type control group (WT). Mice in the TgR group were given a rhubarb decoction by gavage at 0.91 g kg^−1^ every day for 60 consecutive days. Mice in the TgP group were given donepezil solution at 1.5 mg kg^−1^ daily for 60 days. The Tg model group and the WT control group were given 1 ml normal saline every day. The fecal samples of all mice were collected from 8-month-old mice after 3 days of acclimation. After 30-day-treatment, fecal samples of all mice were collected as 9-month-old samples. And after feeding for 60 days, fecal were collected as 10-month-old samples.

### Behavioral Test

#### Morris Water Maze Test

The Morris water maze test was performed as described by Vorhees and Williams (Vorhees and Williams, 2006). The escape latency during the spatial learning phase and the number of platform crossings and the time spent in the target quadrant were recorded. The detailed experiments were provided in the Supplementary Material.

#### Step-down test

The step-down test was performed as described by Ruan et al. (2016). The error times during training, step-down delay and error times during experiments were recorded. The detailed experiments were provided in the Supplementary Material.

### Tissue Collection

#### Brain Tissue Collection

After the behavioral tests, mice were deeply anesthetized with 10% chloral hydrate and euthanized by cervical dislocation. After craniotomy on ice, the whole brain was removed and the hippocampus was quickly separated, weighed, and frozen at −80°C for subsequent enzyme-linked immunosorbent assay (ELISA) and neurotransmitter analyses. Brain tissue samples from mice in each group were also collected, fixed, paraffin-embedded, and sectioned at 5 µm for pathological staining.

#### Fecal Sample Collection

Fecal samples were collected from 8-month-old mice after 3 days of acclimation. After feeding for 30 days, fecal samples were collected from the mice at 9 months of age. Finally, samples were collected at the age of 10 months after feeding for 60 days. Fecal samples of the same mice in the corresponding groups were collected at each time point and six samples from the WT, Tg and TgR groups were used for 16S rRNA gene sequencing and metabolomics analysis.

### Congo Red Staining

The brain tissues of mice in various treatment groups were subjected to Congo Red staining to visualize amyloid plaques. Paraffin-embedded tissue sections were routinely dewaxed into water. The slices were immersed in alkaline solution for 20 min and washed with water. Alkaline Congo Red solution was soaked for 20 min and washed with water. The sections were immersed in alkaline solution for differentiation, observed under a microscope until the tissue staining contrast was clear, then washed with water to stop staining. Harris hematoxylin was applied for 5 min and washed off. Afterward, 1% hydrochloric acid ethanol solution was added for differentiation for a few seconds and the sections were washed with water for 15 min. The sections were dehydrated with gradient ethanol, hyalinized with xylene, and sealed with neutral gum.

### Hematoxylin and Eosin Staining

The brain tissues of mice were fixed with neutral formalin, dehydrated with ethanol, and removed with xylene. The brain tissues were embedded in paraffin and sectioned at 5 μm. The paraffin sections were immersed in 100% xylene twice for 10 min each time and soaked in 95, 80, and 70% alcohol for 3 min in turn. Finally, phosphate-buffered saline (PBS) and distilled water were used to rinse the sections three times, for 2 min each time. The sections were soaked in hematoxylin solution, stained for 10 min, and washed with running water until the water was clear with no purple color. The cells were differentiated with 75% hydrochloric acid ethanol for 3 s (three times) and washed with running water. The nuclei were confirmed to have turned blue under the microscope. Eosin staining was performed for 5 min. The stained sections were dehydrated with pure alcohol and washed with xylene until the cut was transparent and sealed with neutral gum.

### Iba-1 Immunohistochemical Staining

Tissue sections of the whole brain were embedded with paraffin and prepared for immunohistochemical staining. After deparaffinization and rehydration in graded alcohols, antigen retrieval was performed with citrate buffer for 10 min at 90°С. Next, the sections were gradually cooled at room temperature to block endogenous peroxidase activity. The sections were then washed thrice with PBS and blocked with goat serum for 10 min at room temperature. After the goat serum was removed, the sections were incubated with mouse-anti Iba-1 at 4°С overnight, followed by intubation with biotin-labelled goat anti-mouse secondary antibody for 10 min and streptomycin anti-biotin peroxidase for 10 min at room temperature. Then, the sections were labelled with 3,3′-diaminobenzidine, followed by hematoxylin counterstaining. Lastly, after washing, the sections were dehydrated through gradients of ethanol and xylene. PBS displacement of the primary antibody was used as the negative criterion.

### Analysis of Aβ, Aβ_42_, Interleukin-1β, IL-18, and Tumor Necrosis Factor-α Levels with Enzyme-Linked Immunosorbent Assay

Hippocampus samples from the brain were homogenized and the concentrations of Aβ, Aβ_42_, IL-1β, IL-18, and TNF-α were measured using an ELISA kit (BlueGene Biotech, Shanghai, China) according to the instructions. The standard curve was established and used to calculate the levels of Aβ, Aβ_42_, IL-1β, IL-18, or TNF-α in the tissues. The obtained values were corrected for the wet weight of the brain sample and expressed as µg/mg.

### Quantification of Neurotransmitters in Brain Tissue

#### Brain Tissue Sample Processing

The brain tissue was cut into pieces and mixed evenly. Afterward, 0.5 ml of water-methanol (8:2, v/v) was added to every 0.25 g brain tissue to remove protein and tissue homogenate was prepared in a homogenizer. The prepared homogenate was centrifuged twice at 4°C 13709 × g for 10 min. After centrifugation, 200 μl of homogenate supernatant solution was removed and added to 0.8 ml 0.1% formic acid acetonitrile (40–60%) followed by vortexing for 2 min. The prepared solution was centrifuged at 4°C, 13709 × g for 10 min and placed in an injection vial.

#### Liquid Chromatography–Mass Spectrometry Method

Ultra-Performance Liquid Chromatography (UPLC) analysis was performed on a Waters Acquity UPLC system (Waters Corporation, Milford, MA, United States) consisting of a binary solvent system, an autosampler, and a column temperature controller. Chromatographic separation was carried out on an ACQUITY BEH C18 column (2.1 mm × 100 mm, 1.7 μm, Waters, United Kingdom). The mobile phase was composed of eluent A (0.3% formic acid in water) and eluent B (acetonitrile). The line gradient program was optimized as follows: 0–2 min, maintained at 2% B; 2–3 min, increased from 2% B to 30% B; 3–3.5 min, increased from 30% B to 90% B; 3.5–5 min, maintained at 90% B; 5–5.1 min decreased from 90% B to 2% B; 5.1–7 min maintained at 2% B for column equilibrium. The column temperature was set at 30°C and the sample chamber temperature was set at 4°C. The mobile phase flow rate was set at 0.3 ml/min, and the injection volume was 2 μl for each run.

MS data were recorded using the Waters XEVO TQ-S system (Waters Corporation, Manchester, United Kingdom) equipped with an electrospray ionization source in positive ion mode with multiple reaction monitoring (MRM) of the transition of *m/z* 176.9360 → 160.1180 for serotonin (5-HT); *m/z* 146.0780 → 87.0840 for acetylcholine (Ach); *m/z* 154.3020 → 137.1390 for dopamine (DA); *m/z* 103.9040 → 86.9900 for γ-aminobutyric acid (GABA); *m/z* 148.2130 → 84.1240 for glutamate (Glu), and *m/z* 169.9140 → 152.1190 for norepinephrine (NE). The MS parameters were optimized as follows: the ion spray voltage was 4000 V; capillary voltage was 3.0 kV; cone voltage was 30 V; nitrogen was used as the desolvation gas and the cone gas with flow rates of 800 and 150 L/h, respectively; the source and desolvation temperatures were set at 150 and 400°C, respectively; the sheath and auxiliary gas pressures were 20 psi and 10 psi, respectively. Thereafter, the MS/MS conditions were optimized for the internal standard by infusing the individual solution into the electro-spray source. The optimized cone voltage and collision energy were 2 V and 10 eV for 5-HT, 32 V and 12 eV for Ach, 2 V and 8 eV for DA, 22 V and 8 eV for GABA, 2 V and 14 eV for Glu, and 72 V and 6 eV for NE, respectively.

### 16S rRNA Microbial Community Analysis

#### Illumina MiSeq Sequencing

The 16S rRNA sequencing was performed by Majorbio Bio-Pharm Technology Co. Ltd. (Shanghai, China). Purified amplicons were pooled in equimolar and paired-end sequences (2 × 300) on an Illumina MiSeq platform (Illumina, San Diego, United States) according to the standard protocols provided by Majorbio Bio-Pharm Technology Co. Ltd. (Shanghai, China). The detailed method of RNA extraction, PCR amplification and data processing is provided in the Supplementary Material.

#### Data Analysis

Association network analysis was performed using the Co-Net v1.1.1. beta tool (Faust and Raes, 2016) on Cytoscape v3.7.2. (Shannon et al., 2003). Taxa below a sum of 120 and 12 occurrences per condition were discarded and the relative abundances were calculated. Networks were inferred based on the 1000 top and bottom edges for each of the Pearson, Spearman, Bray, and Kullback-Leibler correlation methods with 1000 iterations. The final *p*-values were computed during bootstrapping and adjusted with Benjamini-Hochberg correction for multiple testing.

### Metabolomics Analysis

Hundred-milligram fecal samples from each mouse were weighed and placed in a 2 ml centrifuge tube. Three times the volume of 50% methanol aqueous solution was added for 5 min, and centrifuged at 13709 × g for 10 min. The supernatant was dried with nitrogen and 100 μl of 50% methanol aqueous solution was added for re-dissolution. After shaking for 30 s and standing for 10 min, the supernatant was centrifuged at 13709 × g at 4°C for 10 min and the supernatant was placed in an injection vial.

UPLC analysis was carried out on a Waters Acquity™ UPLC system (Waters Corporation, Milford, MA, United States) consisting of a binary solvent system, an autosampler, and a column temperature controller. Chromatographic separation was carried out on an ACQUITY UPLC^®^ HSS T3 column (2.1 mm × 100 mm, 1.8 μm, Waters, United Kingdom). The mobile phase was composed of eluent A (0.1% formic acid in water) and eluent B (acetonitrile). The line gradient program was optimized as follows: 0–0.5 min, 1% B; 0.5–4 min, 1–20% B; 4–8 min, 20–100% B; 8–9 min, 100% B; 9–9.5 min, 100–1% B; 9.5–11 min 1% B. The column temperature was set at 45°C and the sample chamber temperature was set at 4°C. The mobile phase flow rate was set at 0.3 ml/min and the injection volume was 3 μl for each run.

MS analysis was carried out with a Waters SYNAPT G2-SI MS system (Waters, United States) equipped with an electrospray ionization source. The analysis was performed in the positive and negative ion electrospray modes. The source parameters were set as follows: capillary voltage, 3.0 kV; cone voltage, 28 V; source temperature, 100°C; desolvation temperature, 400°C; the cone gas flow, 35 L/h; desolvation gas flow, 800 L/h. The low collision energy was 6 eV and the high collision energy was 10–65 eV. Mass spectra were recorded across the *m/z* range of 50–1200 and 3D data were collected in the continuum mode. The mass spectrometry data were acquired and processed with Waters MassLynx V4.1 software.

### Data Processing and Multivariate Data Analysis

The UPLC-quadrupole time-of-flight mass spectrometry (UPLC-Q-TOF/MS) data for the fecal samples were imported into Progenesis QI v1.0 (Nolinear Dynamics, Newcastle, United Kingdom) for peak selection and alignment. After normalizing the data using the total ion intensity, a data matrix of interesting features containing the retention time, *m/z* value, and normalized peak intensity was imported into Metaboanalysis 4.0 for principal component analysis (PCA) and partial least-squares discriminant analysis (PLS-DA) and further confirmed using analysis of variance (ANOVA). The differences in the trends were processed with an unsupervised PCA method. Supervised PLS-DA was used to search for interesting biomarkers. Then, the peak height intensities of the differential metabolites were compared with t-tests using statistical software to confirm the biomarker alterations between the WT and Tg groups at the age of 8, 9, and 10 months. A *p* value < 0.05 was set as the threshold. As a small sample set, a pooled quality control (QC) sample containing equal aliquots of all the samples was run at the beginning of the sample queue for column conditions and injected at regular intervals and the end of the run (Want et al., 2010). Mass data acquisition was performed for the evaluation of the sensitivity and stability of instrument performance with regard to mass accuracy, retention time stability, and the coefficient of variation (CV). All samples were kept at 4°C during the analysis.

### Analysis of Fecal Metabolites and Their Metabolic Pathways

Metabolite peaks were assigned based on MS/MS analysis using the MassFragment™ application manager (Waters Corp., Milford, United States). After applying chemically intelligent peak-matching algorithms, the molecular composition of each metabolite and its fragments were structure-matched using available biochemical databases such as HMDB (http://www.hmdb.ca/), Kyoto Encyclopedia of Genes and Genomes (KEGG; http://www.genome.jp/kegg/), LIPIDMAPS (http://www.lipidmaps.org/), and Chemspider (http://www.chemspider.com). The identified metabolites were subjected to KEGG pathway mapping for metabolic pathway analysis. The KEGG database contains a collection of manually curated pathway maps from genomics, transcriptomics, proteomics, and metabolomics and provides molecular interactions and reaction networks.

### HPLC Fingerprinting of Rhubarb Extraction

#### Chromatographic Conditions

Chromatographic analysis was performed on a Thermo UltiMate 3000 HPLC system (Thermo Scientific, United States). The separations were achieved on an Agilent SB-C18 column (250 mm × 4.6 mm, 5 μm) with the column temperature at 40°C. The mobile phases consisted of acetonitrile (A) and an aqueous solution containing 0.05% phosphoric acid (B) using a gradient elution as follows: 0–10 min 96–89% B, 10–25 min 89–87% B, 25–50 min 87–85% B, 50–70 min 85–80% B, 70–100 min 80–67% B, 100–115 min 67–40% B, and 115–140 min 40% B with a flow rate of 1.0 ml/min. The injection volume was 10 μl and the detection wavelength was at 268 nm.

#### Preparation of Reference Compound Solution

Stock solutions were prepared by dissolving seven substances (emodin (BP0532), 1.95 mg; rhein (BP1208), 1.95 mg; chrysophanol (BP0348), 1.51 mg; physcion (BP1092), 1.61 mg; aloe-emodin (BP0146), 1.75 mg; sennoside A (BP1292), 1.50 mg; sennoside B (BP1293), 2.36 mg) in 200 µl dimethyl sulfoxide (DMSO) as described in our previous paper (Gao et al., 2009) and distilled water was added to a constant volume of 5 ml. All the compounds were obtained from Chengdu Biopurify Phytochemicals Ltd. and the purity of these compound is over 98.0%. Subsequently, each reference compound solution was diluted to 100 times with distilled water and the diluent was filtered through a 0.22 μm microporous membrane and placed in an injection vial.

#### Method Validation

The precision was determined by successively analyzing the same sample solution six times. The repeatability was assessed by analyzing six independently prepared sample solutions. The stability was evaluated with the same sample solution at different periods in 1 day (0, 2, 4, 8, 16, 24 h). Each sample solution was tested twice in parallel.

#### Data Analysis

The data analysis was performed on a “TCM chromatographic fingerprint similarity evaluation system (version 2012, Chinese Pharmacopoeia Commission)”.

### RT-PCR

The total bacterial DNA was extracted from the fecal samples of mice in each group with the E.Z.N.A ™. Stool DNA kit (D4015, Omega Biotek, United States), and the procedures were carried out according to the manufacturer’s instructions. First, fecal samples were removed from a −80°C refrigerator, 200 mg of feces from each sample was measured into a 1.5 ml sterile centrifuge tube, and genomic DNA was detected with 2% agarose gel electrophoresis. For detection of the bacteria *Marvinbryantia* (Desai et al., 2016) and *Erysipelatoclostridium* (Zakham et al., 2019), extracted bacterial DNA was subjected to RT-PCR using the CFX Connect real-time PCR system (Bio-rad Laboratories, Hercules California, United States). PCR was performed with a 10 μl sample containing 5 μl SG Green qPCR Mix (with ROX Q1002, SinoGene), 0.2 μl 20 μm upstream and downstream primers, 1 μl bacterial genomic DNA template, and 3.6 μl deionized water. In the blank control, the template DNA was replaced with deionized water. After the PCR reaction, the melting curve temperature was set at 60–95°C and increased by 0.5°C/s. For detection of the bacteria *Bacteroides* (Haugland et al., 2010) and *norank_f_Ruminococcaceae* (Jiang et al., 2017), extracted bacterial DNA was subjected to RT-PCR using the LineGene 9600 Plus real-time PCR system (Bioer Technology, Hangzhou, China). PCR was performed with a 20 μl sample containing 10 μl ChamQ SYBR Color qPCR Master MiX (Vazyme Biotech Co., Ltd, Nanjing China), 0.4 μl 5 μm upstream and downstream primers, 2 μl bacterial genomic DNA template, and 7.2 μl deionized water. In the blank control, the template DNA was replaced with deionized water. After PCR reaction, the melting curve temperature was set at 60–95°C and increased by 0.5°C/s. Table 1 shows the sequences for each primer set, which targeted the 16S rRNA genes for each bacteria group. Each standard curve was prepared based on the cell numbers measured using a bacterial counting chamber with each strain indicated. DNA from each standard strain was extracted as described above and used for RT-PCR. All RT-PCR experiments were performed with duplicates for each sample. The detailed PCR reaction information is listed in the Supplementary Material.

**TABLE 1:** Primer sets used in this study.

Target Bacteria	Sense primer	Anti-sense primer
*Bacteroides*	5′-CAT​GTG​GTT​TAA​TTC​GAT​GAT-3′	5′-AGC​TGA​CGA​CAA​CCA​TGC​AG-3′
*Erysipelatoclostridium*	5′-GAC​ACT​GCA​TGG​TGA​CC-3′	5′-GGT​TTC​TAT​GGC​TTA​CTG-3′
*Marvinbryantia*	5′-CAG​GGA​TTT​TAC​GTG​CTT​TAT​TTT​AGT​TAT-3′	5′-AGT​TCG​GAT​TCG​CTC​GTA​TTT​TCT-3′
*norank_f_Ruminococcaceae*	5′-TGT​TAA​CAG​AGG​GAA​GCA​AAG​CA-3′	5′-TGC​AGC​CTA​CAA​TCC​GAA​CTA​A-3′

### Cell Culture

#### Primary Culture of Rat Neurons

Twenty specific-pathogen-free Sprague-Dawley (SD) neonatal rats were purchased from SPF Biotechnology Co., Ltd (Beijing). The healthy SD neonatal rats were sterilized with 75% alcohol within 24 h after birth and decapitated under sterile conditions. The scalp and skull were cut. Brain tissue was taken out and placed in a dish containing cold D-Hank’s solution with pH 7.2 free of calcium and magnesium. The brain tissue was peeled under sterile conditions and the cerebellum, hippocampus, and medulla were removed. The cortex was isolated and the meninges and blood vessels were carefully peeled off. The tissue was cut into blocks of about 1 mm^3^ using iris scissors, digested in 0.125% trypsin for 20 min at 37°C and shaken two to three times. The supernatant was discarded and complete media was added to terminate digestion. The tissues were rinsed twice and gently pipetted 20 times using a Pasteur pipette. The cell suspension was allowed to stand for 2 min. The cell suspension was collected in a new centrifuge tube and centrifuged at 95 × g at 4°C for 10 min. The supernatant was discarded. The cells were resuspended in complete media and filtered with a 200 mesh stainless steel filter. The filtrate was stained using trypan blue and the cells were counted using a hemocytometer under a microscope. The cells were inoculated at an intensity of 8.0 × 10^4^ to 1.0 × 10^5^ ml with 100 μl/well into a 96-well plate pre-coated with poly-L-lysine. The cells were cultured for 4–6 h in an incubator at 37°C with 5% CO_2_ and the medium was replaced with serum-free media. After culturing for 3 days, Ara-C working solution was added to inhibit over-proliferation of non-neuronal cells and aspirated after 24 h. Thereafter, half of the medium was replaced every 3 days. Cells collected from days 7–21 of culture were used for the experiment.

### Cell Culture of Rat Microglial Cells

The murine microglial cell line BV-2 was cultured in high-glucose Dulbecco’s modified Eagle’s medium (DMEM) supplemented with 10% fetal bovine serum (FBS) and 1% penicillin/streptomycin (P/S) in a humidified incubator with 5% CO_2_ at 37°C. BV-2 cells were plated into 96-well plates (1.0 × 10^5^ cells/well) and incubated overnight for subsequent experiments.

### MTT Analysis

The 3-(4,5-dimethylthiazol-2-yl)-2,5-diphenyltetrazolium bromide (MTT) assay is based on the protocol described by Mosmann (1983). The assay was optimized for the cells used in the experiments. Briefly, after the cells were incubated with different concentrations of *o*-tyrosine (*o*-tyr) (0.5, 1, and 2 mM) for 48 h at 37°С (Pennathur et al., 1999), the culture medium was removed, then the cells were incubated for 4 h with 0.5 mg ml^−1^ of MTT and dissolved in serum free medium. Washing with PBS was followed by the addition of 100 µl DMSO and gentle shaking for 10 min to facilitate complete dissolution. After the formazan crystals had dissolved, the absorbance was determined spectrophotometrically at 490 nm on an ELX800 UV universal microplate reader. The results were analyzed with the Soft max pro software (version 2.2.2) and are presented as a percentage of the control values.

### Active Oxygen Detection

Reactive oxygen species (ROS) production in neuronal and BV-2 cells was assessed with a 2′,7′-dichlorodihydrofluorescein diacetate (DCFH-DA) probe. Different concentrations of *o*-tyr (0.5, 1, and 2 mM) were incubated with the cells for 48 h at 37°С. After the culture medium was removed, the cells were treated with 5 µM DCFH-DA at 37°С for 30 min. Washing with PBS three times was followed by the addition of 100 µl PBS and the intracellular ROS levels in the cells were viewed with a fluorescence microscope (Nikon Eclipse).

### Measurement of Nitric Oxide

Nitric oxide (NO) production in neuronal and BV-2 cells was performed using 2′,7′-dichlorodihydrofluorescein diacetate (DAF-FM DA). Different concentrations of *o*-tyr (0.5, 1, and 2 mM) were incubated with the cells for 48 h at 37°С. After the culture medium was removed, DAF-FM DA (5 µM) was added to the wells. After incubating at 37°С for 20 min, the chemical was removed and the cells were washed three times with PBS followed by measurement with a fluorescence microscope.

### Statistical Analysis

Statistical analyses were performed using R 2.15.0 and GraphPad Prism software v 8.0. All the data were presented as the mean ± SEM. The significance of the differences between two groups was analyzed using the Student’s unpaired *t*-test and multiple comparisons were analyzed using one-way ANOVA followed by Dunnett’s post hoc test. The differential abundances of genera and metabolites were determined using non-parametric tests including the Wilcoxon rank sum test and Mann–Whitney U test. The correlations among fecal metabolites, 16S levels, and physiological and biochemical indexes were tested with both the Pearson Correlation Coefficient and Spearman rank correlation. *p* values were corrected for multiple comparisons using the Benjamini–Hochberg false discovery rate (FDR) and *p* < 0.05 was statistically significant.

## Results

### Rhubarb Alleviates Cognitive Impairment in APP/PS1 Transgenic Mice

**FIGURE 1:**
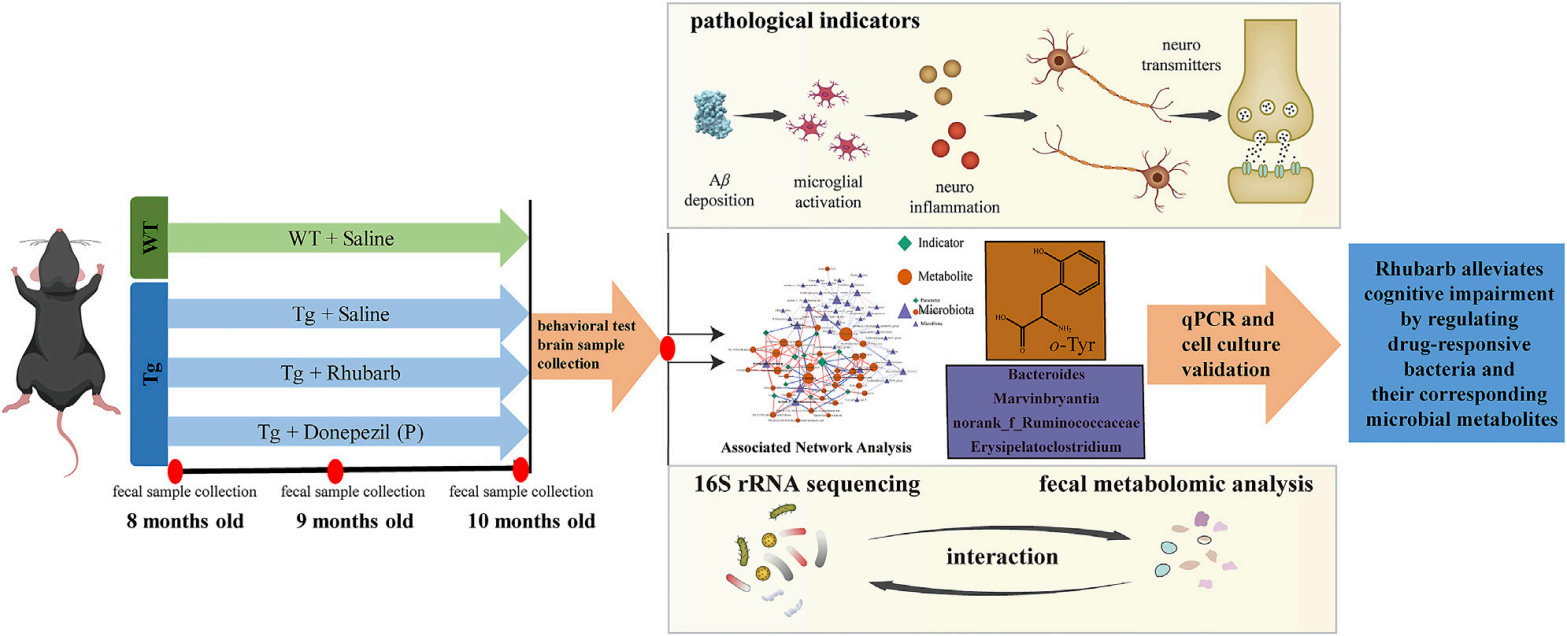
The main content of this study.

**FIGURE 2:**
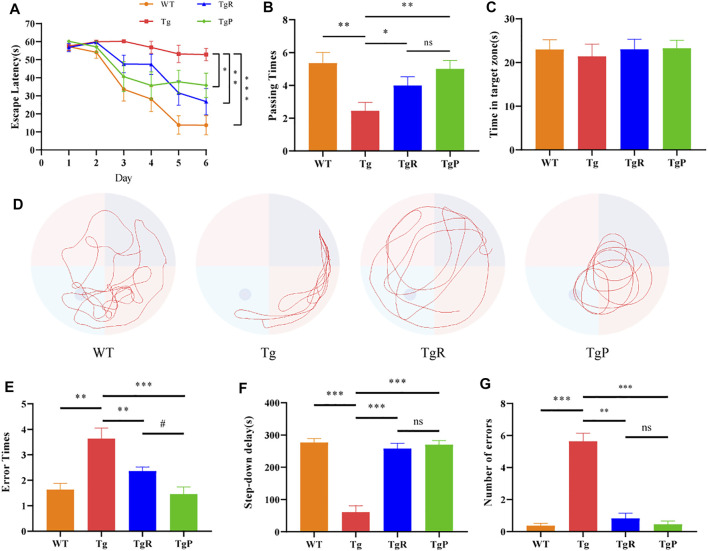
Effects of rhubarb on memory and cognitive function of APP/PS1 mice. **(A–D)** Morris water maze test, including assessments of escape latency **(A)**, platform crossing times **(B)**, swimming time in the target quadrant **(C)**, representative swimming path **(D)**. **(E–G)** Step-down test, including assessments of error times during training **(E)**, step-down delay **(F)**, error times during experiments **(G)**. Values are expressed as the mean ± S.E.M.; *n* = 9, *p < 0.05 versus Tg, #*p* <0.05 versus TgP, by Student’s unpaired *t*-test.

Our previous study revealed that *Rheum tanguticum* ameliorated cognitive impairment in AD rat model, possibly due to the regulation of the gut microbiota (Zhao et al., 2019). In this study, to further verify the therapeutic effects for the treatment of AD, rhubarb extraction with a uniform and stable quality (Supplementary Figure S1A) and donepezil as positive drug were administrated to APP/PS1 transgenic mice for 2 months, named the TgR and TgP group, respectively. The main content of this study was shown in Figure 1. The effect of rhubarb on the learning and memory abilities of the Tg mice was tested by the Morris water maze. During the training period, the escape latencies of the mice in each group were similar on the first day, and gradually decreased in the next 5 days (Figure 2A). On the sixth day, the escape latency of Tg mice was significantly longer than that of wild-type (WT) mice. After the treatment of rhubarb or donepezil, the escape latency of Tg mice was significantly shortened and there was no significant difference between them. In the probe trial, Tg mice exhibited a lower platform passing times (Figure 2B) and shorter swimming time in target zone (Figure 2C). However, rhubarb or donepezil treatment significantly reversed these defects in Tg mice. These results indicated that rhubarb could attenuate spatial learning and memory deficits in APP/PS1 mice (Figure 2D).

The step-down test was performed to investigate the passive avoidance ability of Tg mice. Compared with the WT mice, Tg mice showed more errors times during the training period (Figure 2E) and shorter step-down delay (Figure 2F) accompanied by a greater number of errors (Figure 2G) during the experimental period. Fortunately, rhubarb or donepezil treatment reduced the errors times during the training period, increased the step-down delay, and reduced the number of errors during the experimental period in Tg mice with no significant difference. These results suggested that rhubarb extract could alleviate the stimulation avoidance response and cognitive impairment in APP/PS1 mice.

### Rhubarb Reverses the Pathological Changes in Tg Mice

**FIGURE 3:**
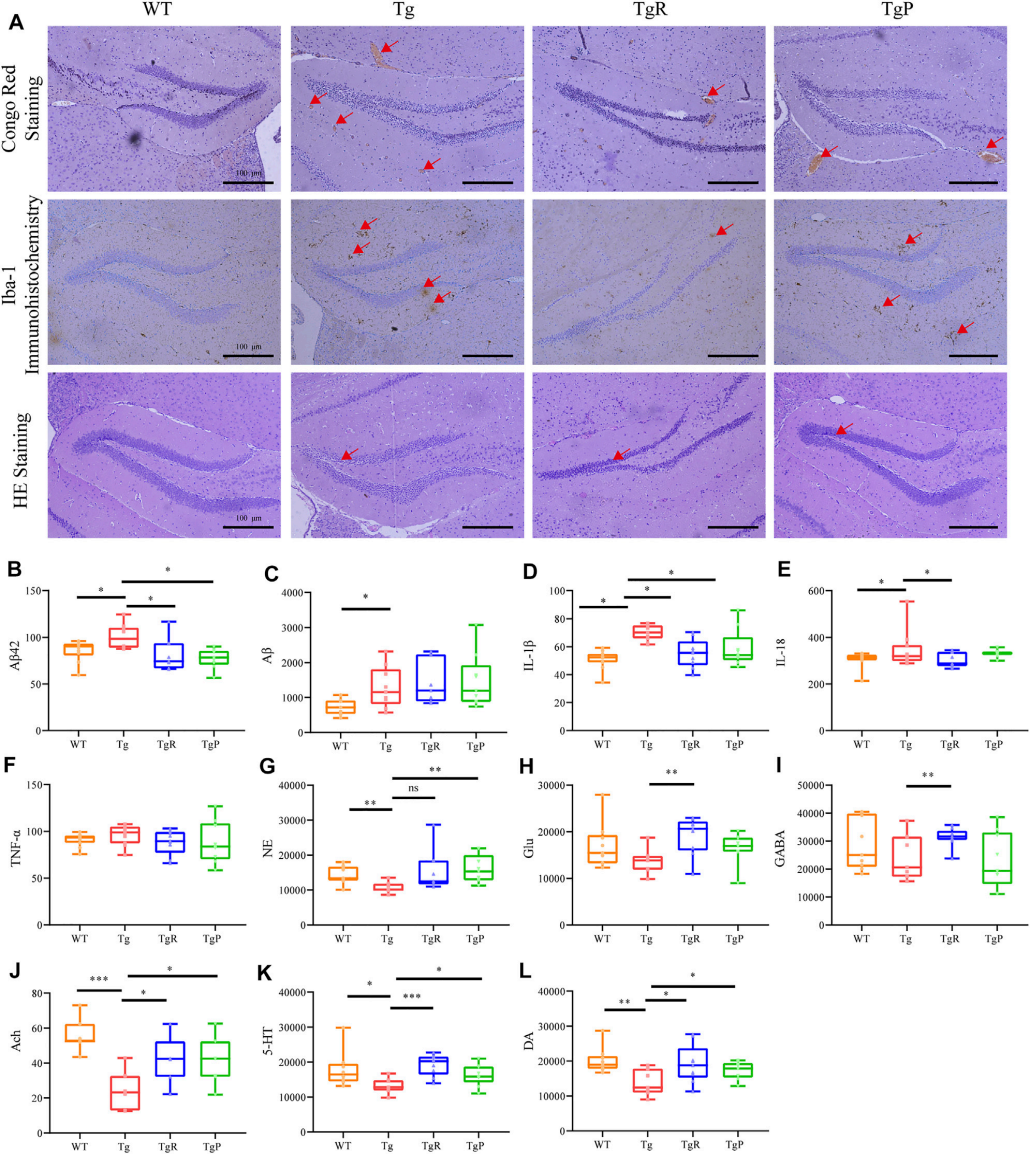
Rhubarb alleviates the pathological changes in APP/PS1 mice. **(A)** Histopathology examination of the hippocampus in the brain. (Scale bar: 100 μm). **(B–L)** Levels of β-amyloids, pro-inflammatory cytokines, and neurotransmitters in the brain. Aβ_42_ (B), Aβ **(C)**, IL-1β **(D)**, IL-18 **(E)**, TNF-α **(F)**, norepinephrine (NE) **(G)**, glutamate (Glu) **(H)**, γ-aminobutyric acid (GABA) **(I)**, Acetylcholine (Ach) **(J)**, serotonin (5-HT) **(K)**, dopamine (DA) **(L)**. Values are expressed as the mean ± S.E.M.; *n* = 9; *p < 0.05 versus Tg; **p < 0.01 versus Tg; **p < 0.001 versus Tg, by Student’s unpaired *t*-test.

After the behavioral tests, the mice were sacrificed to further investigate the effect of rhubarb on the pathological changes in the brain of Tg mice, including Aβ deposition, activated microglia, neuronal damage and neurotransmitter disorders. The main pathological feature of AD is senile plaques composed of extracellular Aβ, which mainly deposited in the brain parenchyma and cerebral vessels (Pistollato et al., 2016; Friedland and Chapman, 2017). Here, Aβ deposition in the brain of Tg mice was stained by Congo Red, and the amount of total Aβ and Aβ_42_ were quantified by ELISA. As expected, cerebral Aβ deposition (Figure 3A), and the levels of hippocampal Aβ and Aβ_42_ (Figures 3B,C) in Tg mice was significantly higher than that of WT mice. After the treatment of rhubarb or donepezil, Aβ deposition in the brain of Tg mice was significantly reduced (Figure 3A). Moreover, although there was no significant difference in the level of total Aβ between the TgR and Tg group, the level of Aβ_42_ was markedly reduced in the TgR group (Figures 3B,C). Donepezil treatment significantly reduced both the levels of total Aβ and Aβ_42_.

Recent studies have shown that Aβ deposition in the brain can activate microglia and release inflammatory factors (Wu Y. et al., 2019). Therefore, the activation of microglia in the brain and the content of inflammatory factors in the hippocampus were evaluated by Iba-1 immunohistochemistry and ELISA, respectively. Compared with the WT mice, both the density and the number of activated microglia in the hippocampus of Tg mice were larger (Figure 3A). In addition, the levels of IL-1β and IL-18 in Tg mice were significantly higher than that of WT mice, and the level of and TNF-α was slightly higher (Figures 3D–F). As shown in Figure 3, rhubarb treatment exhibited a significant decrease in the number of activated microglia, and the levels of IL-18 and IL-1β (Figure 3) alone with a slight decrease of TNF-α similar to the donepezil treatment.

Activated microglia release inflammatory factors and induce neuroinflammation reaction, and eventually lead to neuronal apoptosis, resulting in the decline of learning and memory abilities (Rothhammer et al., 2018; Wu Y. et al., 2019). Therefore, the morphology of neurons in the brain tissue of mice was observed by HE staining. The results showed the disordered arrangement of neurons, condensed cytoplasm and karyopyknosis, and decreased number of neurons were observed in Tg mice (Figure 3A). Rhubarb or donepezil treatment reversed the above histopathological changes.

Neuronal injury can lead to the neurotransmitter disorders in the brain. Different kinds of neurotransmitters participate in neural activities in multiple brain regions. Therefore, the levels of 5-HT, DA, NE, Ach, Glu, and GABA in the brain of mice were detected by UPLC-TQ/MS. Compared with the WT mice, the levels of NE, Ach, 5-HT, and DA in the brain of Tg mice were significantly decreased, and the levels of Glu and GABA were slightly reduced (Figures 3G–L). As illustrated in Figure 3A, rhubarb and donepezil showed a favourable regulation in neurotransmitters in brain of Tg mice. Altogether, these results provided evidence that rhubarb could alleviate the pathological changes in Tg mice. Moreover, after 60 days treatment with rhubarb, there were no obvious abnormalities in body weight (Supplementary Figure S2A) and also no tissue damage or any other adverse effect in brain tissues (Supplementary Figure S2B).

### Screening Drug-Responsive Bacteria in Rhubarb Treated Tg Mice in an Age-dependent Alteration by RT-PCR Analysis

**FIGURE 4:**
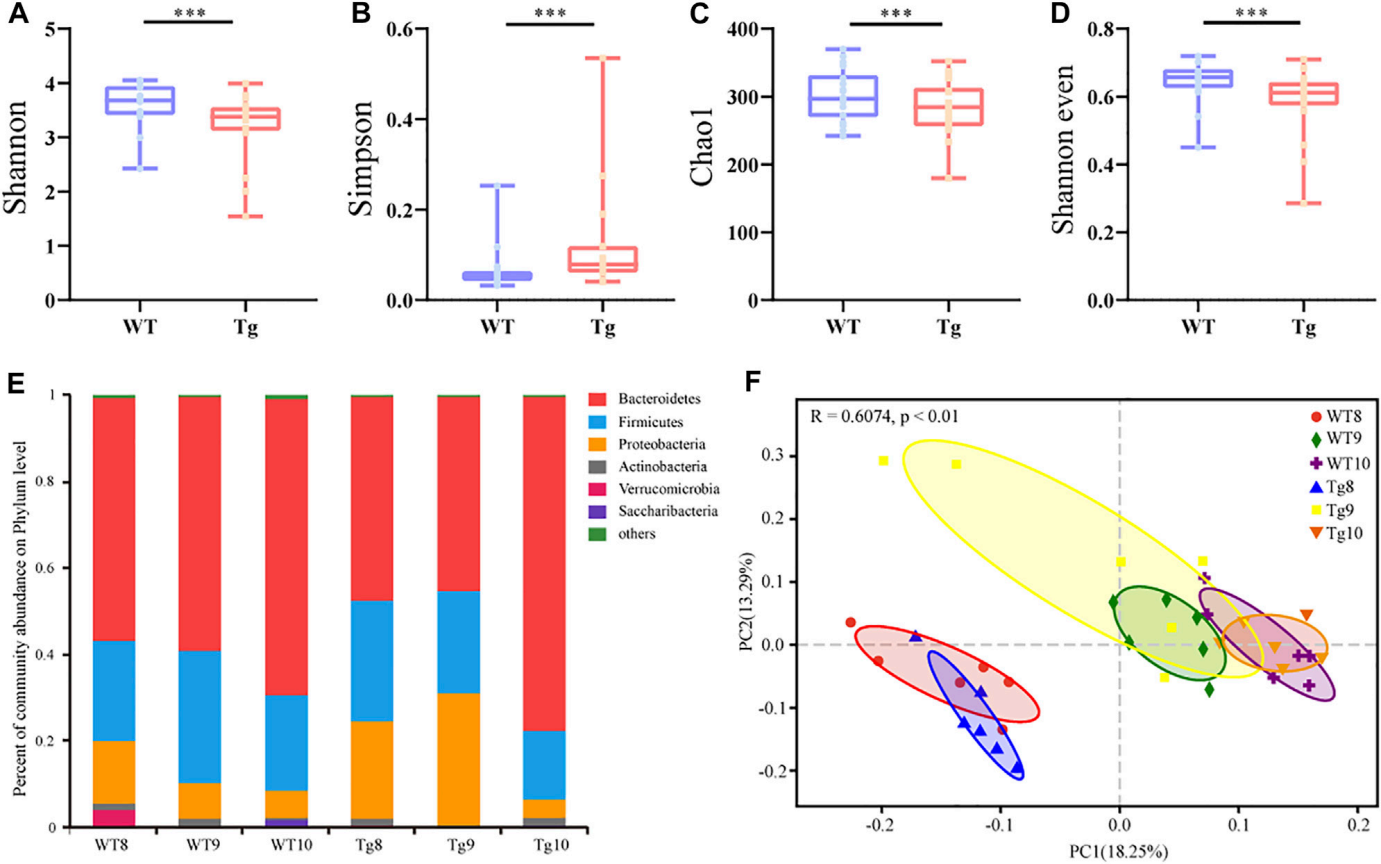
Alteration of gut microbiota profile in AD model **(A–D)** α diversity of WT (*n* = 6) and Tg mice (*n* = 6) evaluated by Shannon **(A)**, Simpson **(B)**, Chao1 **(C)**, and Shannon even indices **(D)**. Error bars represent mean ± S.E.M. *p < 0.05 versus Tg; **p < 0.01 versus Tg; **p < 0.001 versus Tg, ***p < 0.0001 versus Tg, by Wilcoxon rank-sum test. **(E)** Bacterial taxonomic profiling at phylum level. Number in the group name represents months of age. **(F)** Principal component analysis (PCoA) based on unweighted UniFrac distances.

In order to explore the changes in intestinal microbiota during the progression of AD, gut microbial dysbiosis in Tg mice in an age-dependent alteration (six samples per time point, *n* = 36) was evaluated, and distributed intestinal microbiota was identified by 16S rRNA gene sequencing. As a result, a total of 1237622 valid sequences were obtained from 36 samples (average 34378 ± 9394 reads per sample). These sequences were classified into 521 OTUs with a 97% similarity level. The α-diversity index includes the Chao1, Shannon, and Simpson indexes, which were used to determine the ecological diversity of the microbial community. The results showed that the α-diversity of gut microbiota in Tg mice decreased to a certain extent compared with the value of WT mice (Figures 4A–D). The dominant microbiota at the phylum level in the WT mice changed regularly with age (Figure 4E).

The *β* diversity of all bacteria in the six groups was calculated by the unweighted UniFrac metric and visualized by principal coordinate analysis, respectively. We found that the gut microbiota composition between WT and Tg mice changed similarly with time, but the variation in Tg mice was more significant (Figure 4F).

**FIGURE 5:**
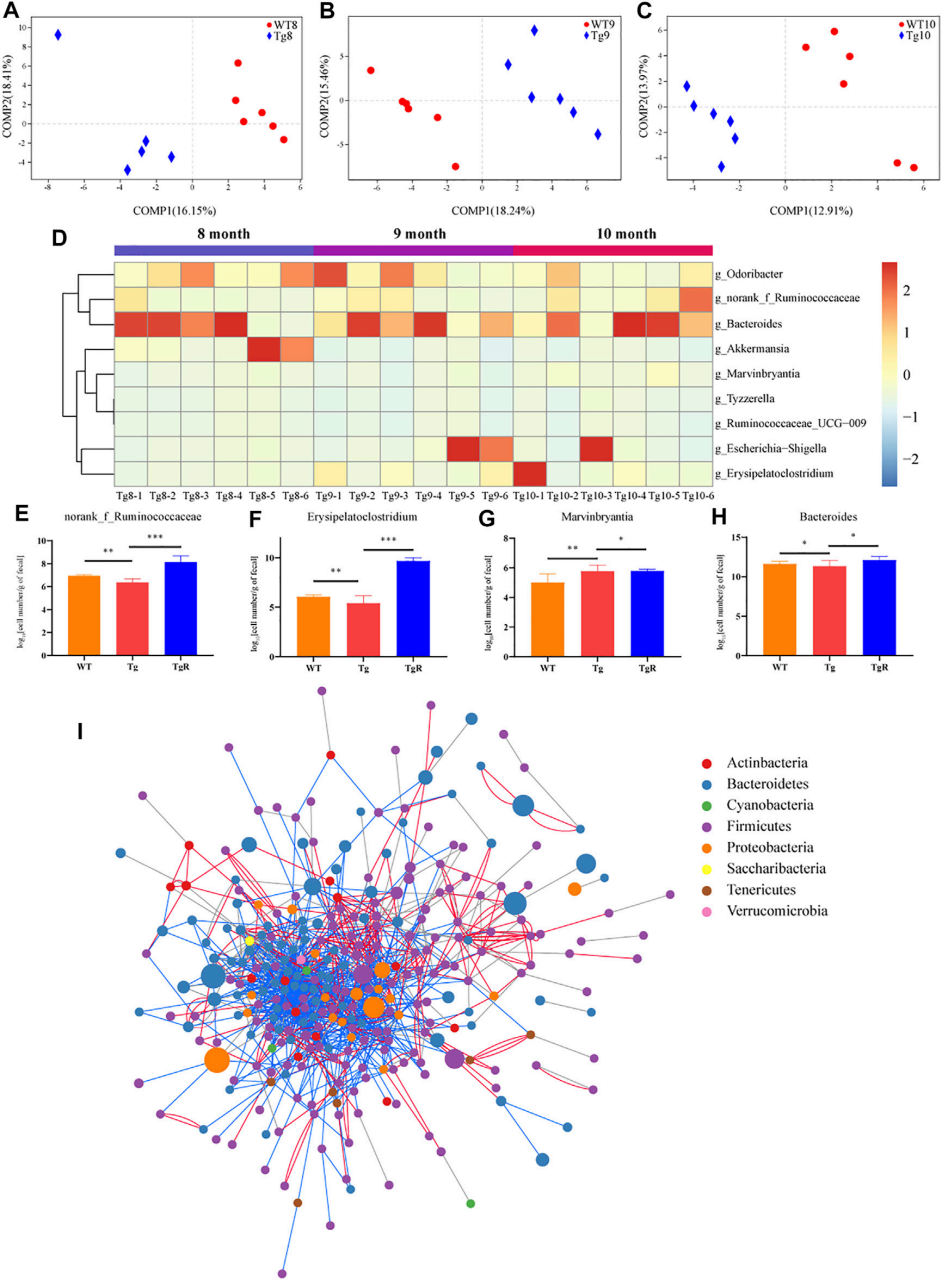
The gut microbiota of Tg mice exhibited a common trend with age. **(A–C)** PLS-DA score plots of WT and Tg groups. Number in the group name represents months of age. **(D)** Heatmap formed by the abundance of genera. **(E–H)** Abundance of the key bacteria in the network, quantified by species-specific quantitative PCR. *p < 0.05 versus Tg; **p < 0.01 versus Tg; **p < 0.001 versus Tg, ***p < 0.0001 versus Tg, by Wilcoxon rank-sum test. **(I)** Co-network constructed by all OTUs in the fecal samples of Tg mice at three time points. The size of the node indicates the relative OTU abundance; the color of the node indicates different phyla; the lines between the nodes indicate co-abundance (red) or co-exclusion (blue) between the nodes.

To further explore the microbiota that was related to the aggravation of the disease, the gut microbiota in WT and Tg mice was compared based on a PLS-DA model. According to the VIP > 0.7, 72 differential bacterial genera were screened at 8 months of age (Figure 5A), 76 at 9 months of age (Figure 5B), and 69 at 10 months of age (Figure 5C), respectively. However, only nine genera exhibited the same trends at the three time points. In the Tg group, the relative abundances of *Tyzzerella*, *Ruminococcaceae_UCG_009*, *Bacteroides*, *Escherichia-Shigella,* and *Marvinbryantia*, *norank_f_Ruminococcaceae* and *Erysipelatoclostridium* increased gradually, while *Odoribacter* and *Akkermansia* decreased gradually (Figure 5D).

After that, drug-responsive bacteria between the Tg and TgR group were identified by RT-PCR analysis. As shown in Figures 5E–H, rhubarb treatment increased the number of *norank_f_Ruminococcaceae*, *Erysipelatoclostridium* and *Bacteroides* and reduced the number of *Marvinbryantia* compared with the Tg group. To confirm the changes in the gut microbiota in Tg mice with age, co-network analysis was conducted on all OTUs detected in fecal samples from Tg mice at three time points (Figure 5I, Supplementary Figure S3). The results showed that there were close interactions between the intestinal microbiota, which increased or decreased together.

### Screening Microbial Metabolites in Rhubarb Treated Tg Mice in an Age-dependent Alteration by Metabolomics Analysis

**FIGURE 6:**
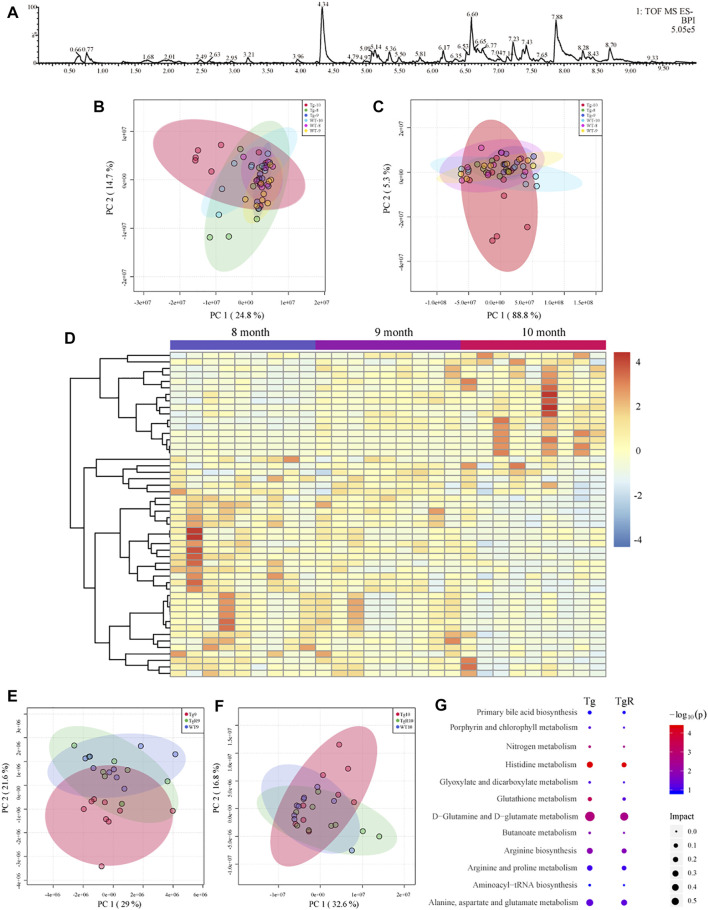
Fecal metabolomics profile of Tg mice **(A–D)**. **(A)** Representative base peak ion chromatogram (BPI) of fecal metabolites from Tg sample. PCA score plots in positive **(B)** and negative **(C)** ion mode. **(D)** Heatmap showing the trend of metabolomic profiling in all the Tg groups. Effects of rhubarb on the fecal metabolomics of AD model **(E,F)**. PCA score plots based on fecal metabolic profiles under the negative ion mode. TgR mice received rhubarb intervention 30 days **(E)** and 60 days **(F)**. **(G)** Pathway enrichment of different metabolites in WT and Tg mice and the metabolites recovered after rhubarb administration. The size of the node represents the path impact value and the colour represents the −log_10_ (*p*) value of the pathway.

To clarify the effects of gut microbiota changes on AD-related pathological changes in mice, the metabolic profiles of fecal metabolites from the Tg and WT groups at 8, 9, and 10 months of age were analysed by UPLC-Q-TOF/MS in positive and negative ion modes. BPIs of the metabolomic profiles of Tg sample in the negative ion modes is shown in Figure 6A. A total of 9941 features in the positive ion mode and 5139 features in the negative ion mode were detected in all samples from the WT and Tg groups.

The fecal metabolic profiles of the WT and Tg mice were analysed with PCA. Different metabolites were observed among the groups at 8, 9, and 10 months of age. The PCA scores for the WT and Tg at three time points are shown in Figure 6B (positive ion mode) and Figure 6C (negative ion mode). It can be observed that the profile for the fecal metabolites showed age-dependent changes. The obvious difference between the WT and Tg groups indicated that the fecal metabolites of Tg mice had changed significantly. To explore potential biomarkers related to the pathogenesis of AD, the fecal metabolomics data for WT and Tg mice was compared by a PLS-DA model (Supplementary Figure S4). According to PLS-DA model with a VIP value greater than 1 and a *p* value less than 0.05 for the ANOVA *t*-test, differential metabolites were identified based on the fragment information and accurate mass number. Through a search and comparison of the HMDB, Metlin, and Chemspider databases, 50 differential metabolites with similar trends were found in the feces of Tg mice at three time points (Figure 6D). We speculate that some of these 50 differential metabolites were directly related to the pathological process in AD.

Drug-specific responsive microbial metabolites in the TgR group were screened by metabolomics analysis. After the administration of rhubarb extract for 30 and 60 days, the fecal metabolic profile for Tg mice showed a gradual recovery to a profile similar to that of WT mice (Figures 6E,F, Supplementary Figures S1B,C). Further, 27 of the 50 metabolites (Figure 6D) present at both time points in the TgR group showed a rhubarb intervention-dependent trend.

To gain insight into the mechanism underlying the rhubarb-induced improvement of cognitive impairment in AD mice, the metabolic pathways for 50 age-dependent metabolites with differences between the WT and Tg groups and 27 biomarkers for the long-term effects of rhubarb on AD cognitive impairment were evaluated. The results showed that the main metabolic pathways for rhubarb were histidine metabolism, D-glutamine, and D-glutamic acid metabolism, aminoacyl tRNA biosynthesis, alanine, aspartic acid, and glutamic acid metabolism (Figure 6G).

### Screening Microbial Metabolites Associated With Rhubarb-Responsive Bacteria by Co-network Analysis

**FIGURE 7:**
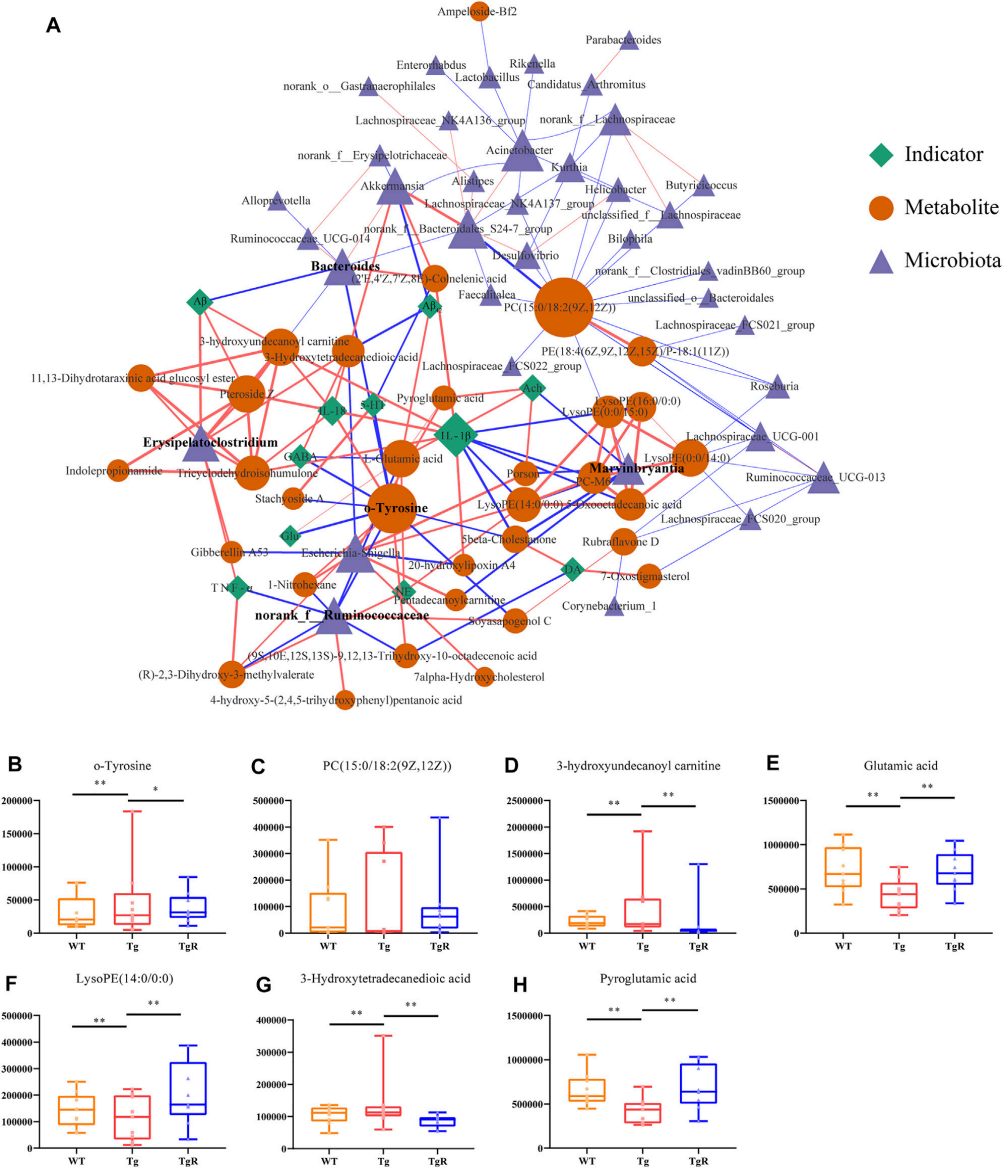
Rhubarb regulates the microbial-metabolite-indicator network of AD model. **(A)** Correlation network for microbiota, metabolites, and pathological indicators for Tg mice. The blue triangle represents bacteria, the yellow circle represents metabolites, and the green diamond represents physiological and biochemical indicators. Lines between nodes indicate positive (red) or negative (blue) correlations. The width of the line indicates the correlation value. **(B–H)** Rhubarb regulates abundance of key metabolites in the network quantified by peak areas. Values are expressed as the mean ± S.E.M.; *n* = 9; *p < 0.05 versus Tg; **p < 0.01 versus Tg; **p < 0.001 versus Tg, by Student’s unpaired *t*-test.

To further screen the microbial metabolites associated with bacteria in response to rhubarb, an improved co-network analysis was conducted based on the gut microbiota data at the OTU level and 50 differential metabolites detected in mouse fecal samples, with five pathological indicators and six neurotransmitters. As shown in Figure 7, *Bacteroides*, *Marvinbryantia*, *norank_f_Ruminococcaceae,* and *Erysipelatoclostridium* were significantly correlated with several biomarkers and pathological indicators. Phosphatidylcholine (PC; 15:0/18:2(9Z,12Z)), *o-*tyr, 3-hydroxyundecanoyl carnitine, L-glutamic acid, LysoPE (14:0/0:0), 3-hydroxytetradecanedioic acid, and pyroglutamic acid showed strong correlations with both the microbiota and indicator nodes. Interestingly, *o-*tyr showed a significant correlation with both the microbiota and indicator nodes (Supplementary Table S6), suggesting that *o-*tyr may lay on an important node in this network. We speculated that these four genera and their related metabolites and pathways may influence AD-related pathological indicators and play an important role in the progression of AD. A variety of pathological indicators for AD are related to intestinal microbiota disorders and metabolic disorders (Supplementary Figure S5). It suggested that the decline of cognitive function in AD was related to the microbiota composition and metabolites and further aggravates cognitive impairment.

As shown in Figure 7, some bacteria and metabolites play pivotal roles in the microbiota-metabolite-indicator network and may react specifically to rhubarb. To understand the potential effect of rhubarb on the microbiota-metabolite-indicator network, metabolite nodes with higher node degrees were analysed. Interestingly, the abundance of *o-*tyr, 3-hydroxyundecanoyl carnitine, L-glutamic acid, LysoPE (14:0/0:0), 3-Hydroxytetradecanedioic acid, and pyroglutamic acid, which connected the microbiota and indicator nodes in the network, were recovered after rhubarb treatment (Figures 7B–H).

### Validation of *o*-tyr as Key Microbial Metabolites Mediating the Pathological Changes in Alzheimer’s disease

As mentioned above, the relative peak area of *o-*tyr in the feces of the Tg group continued to increase with age. The level of *o-*tyr was significantly reduced by rhubarb intervention (Figure 7B). In the microbiota-metabolites-indicators network, *o-*tyr was significantly correlated with two types of bacteria, four pathological indicators, and seven metabolites, suggesting it may play a pivotal role in connecting the microbiota to the pathological indicators in the microbiota-metabolites-indicators network.

**FIGURE 8:**
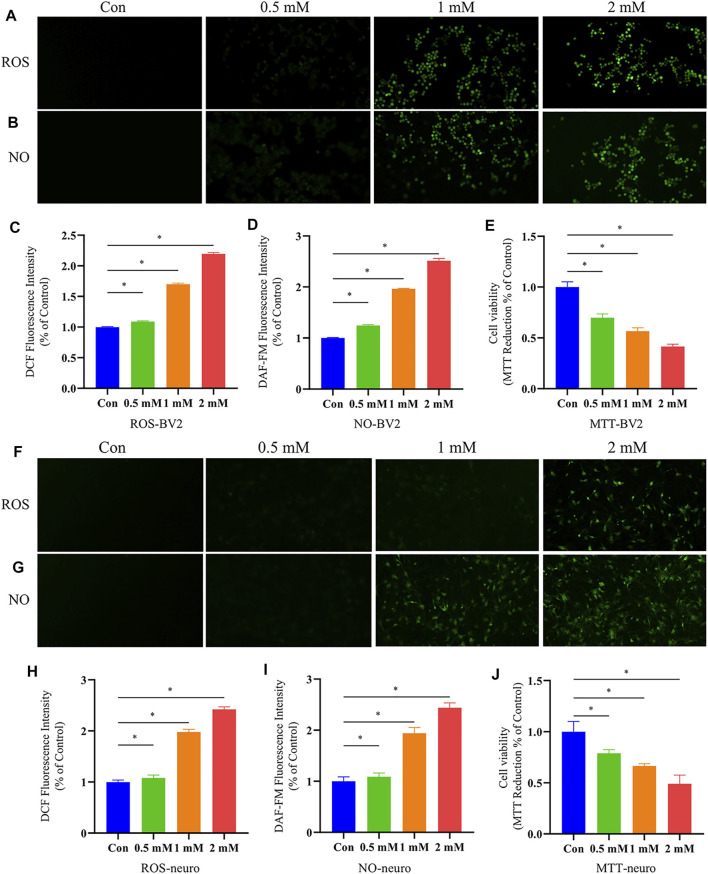
Effects of *o*-tyr on oxidative stress and cell viability of BV-2 and neurons. **(A,B)** Fluorescent images of ROS **(A)** and NO **(B)** in BV-2 cells, with *in situ* signals quantified in **(C)** and **(D)**. **(E)** Effect of *o*-tyr on the viability of BV-2 cells. **(F,G)** Fluorescent images of ROS **(F)** and NO **(G)** in BV-2 cells, with *in situ* signals quantified in **(H)** and **(I)**. **(J)** Effect of *o*-tyr on the viability of neuronal cells. Values are expressed as the mean ± S.E.M.; *n* = 6; *p < 0.05 versus Con, by Student’s unpaired *t*-test.

It has been reported that *o*-tyrosine could not only produce intracellular hydroxyl radicals, but other reactive oxygen species (ROS), such as hydrogen peroxide and superoxide radical anion (Ipson and Fisher, 2016). We speculated that in the process of aging, *o-*tyr from bacteria could enter the brain due to the increased permeability of the blood-brain barrier, induced neuronal oxidative stress and apoptosis. In this study, *in vitro* experiments on primary neurons and BV-2 cells were performed to validate whether *o-*tyr could promote the pathology of AD. ROS is considered to participate in the pathogenesis of AD, which can cause oxidative stress and trigger damages to cells in the brain. The intracellular ROS levels in neuronal and BV-2 cells were determined with DCFH-DA and the fluorescence intensity was further monitored by fluorescence microscope. As shown in Figures 8A,C,F,H, as the increase of *o-*tyr concentration, the fluorescence intensity of both cells significantly enhanced.

NO, as an inflammatory factor, plays an important role in neuronal death. To investigate the effect of *o-*tyr on the inflammatory response in neuronal and BV-2 cells, DAF-FM DA was used as a probe for NO. As shown in Figure 8, the fluorescence intensity in both neuronal and BV-2 cells significantly enhanced with the increase of the *o-*tyr concentration.

Studies have shown the potential of *o-*tyr to induce apoptosis. Therefore, the impact of *o-*tyr on the viability of neuronal and BV-2 cells was evaluated by the MTT assay. As shown in Figure 8, both of the two cells treated with 2 mM *o-*tyr reduced the cell viability to approximately 50%. These results demonstrated that *o-*tyr adversely affected the function and viability of the neuronal and BV-2 cells.

## Discussion

In this study, we found that there were age-dependent alterations in both gut microbiota and fecal metabolites in Tg mice, indicating a dynamic change in microbial community and related metabolites during the development of AD. Through a more accurate co-network analysis based on indicator and multi-omics correlation analyses, we found a significant correlation between the metabolites and microbiota with the development of AD disease. *In vitro* experiments were conducted to confirm the toxicity of the microbial metabolite *o-*tyr, which was closely related to the microbiota and pathological indicators in response to rhubarb intervention. The therapeutic mechanisms by which rhubarb acts on key gut microbiota, affects the related metabolites, and improves pathological indicators and finally cognitive impairment in Tg mice were further examined.

It has been reported that APP/PS1 mice show amyloid deposition at 2 months of age, amyloid plaques at 5 months of age, synaptic loss after 7–9 months, and severe cognitive impairment (Want et al., 2010). In addition, age-dependent alterations in the microbiome of APP/PS1 Tg mice have been reported (Shen et al., 2017; Bauerl et al., 2018). These reports indicate that pathological changes in AD are a dynamic process; hence, disturbed intestinal bacteria based on a single time point are not enough to reflect the pathological process of AD. Accordingly, our study confirmed that the gut microbiota community structure changed with the age of the Tg mice by 16S rRNA analysis. The abundance of *Akkermansia* in the Tg group decreased significantly with the increase of age and was negatively correlated with the Aβ_42_ content in the hippocampus. As a next generation probiotic (Zhang T. et al., 2019), *Akkermansia muciniphila* is considered the most abundant mucolytic bacteria in a healthy gut (Belzer and de Vos, 2012). The continuous decrease of *Akkermansia* in the gut during aging may lead to the thinning of intestinal mucosa and the weakening of the intestinal barrier function, and subsequently the translocation of endotoxins and other proinflammatory bacterial products (Bodogai et al., 2018). The abundance of *Escherichia-Shigella* in the Tg group also increased with age. A clinical study showed that the abundance of *Escherichia-Shigella* involved in inflammatory response increased in the feces of AD patients and there was a significant positive correlation between the expression of IL-6, cxcl2, and NLRP3 (Cattaneo et al., 2017). Interestingly, both *Ruminococcaceae_UCG_09* and *norank_f_Ruminococcaceae* belong to the Ruminococcaceae family, although the two species are similar at the taxonomic level, they may perform distinct functions *in vivo* to influence disease progression (Liu H. et al., 2019). One study reported that high salt diet induced an increase in the abundance of *Ruminococcaceae_UCG_09* in hypertensive mice, accompanied by increased intestinal permeability and inflammation in the small intestine and periphery (Zhang Z. et al., 2019). The increased intestinal permeability led to intestinal bacterial translocation, and the Aβ peptides and LPS secreted by harmful bacteria further induced peripheral and neuronal inflammation, exacerbating cognitive impairment in AD (Friedland and Chapman, 2017). Taken together, our results suggested that there were highly dynamic changes and extensive interactions between the gut microbiota of Tg mice that exacerbated the disease phenotype over time.

Changes in the composition and function of the gut microbiota can affect the overall effectiveness of the drug (Wu TR. et al., 2019; Zeng et al., 2020). Meng Yu et al. (Yu et al., 2017) reported a reduced abundance of *Marvinbryantia* in the gut microbiota and fecal metabolites of rats in a depression model and a significant association existed in bile acid and tryptophan metabolism as well as 5-HT, DA, and NE in the brain. An observational study of AD patients indicated that bile acids may be a biomarker for the early diagnosis of AD (MahmoudianDehkordi et al., 2019). Gut microbiota and the host co-regulate tryptophan metabolic pathways associated with several neurodegenerative diseases (Platten et al., 2019; Dong et al., 2020). Gut microbiota has a regulatory effect on 5-HT production by intestinal enterochromaffin cells and affect the overall tryptophan metabolism and 5-HT levels in the colon and blood, which in turn affects the central concentration of the precursor substance of 5-HT, 5-HTP, and regulates neurotransmitter levels in the brain (Kwon et al., 2019). Herein, rhubarb-responsive bacteria were screened by RT-PCR analysis. We found that the abundance of *Marvinbryantia* gradually decreased with increasing age in Tg mice and was correlated with various metabolites in feces as well as the 5-HT, DA, Glu, and GABA neurotransmitters in the brain. We also found that long-term intervention with rhubarb elevated the abundance of *Marvinbryantia*. Therefore, we hypothesized that *Marvinbryantia* might be an important microbiome target for rhubarb to alleviate AD cognitive impairment.

Studies have indicated that microbial metabolites derived from intestinal bacteria may be important messengers for bidirectional cross-talk between the gut and brain. In food and the human body, choline mainly exists in the form of phosphatidylcholine (PC) (Wang et al., 2011). Many intestinal bacteria that can utilize choline to convert PC into free choline, which is metabolized to trimethylamine (TMA) (Chittim et al., 2019). Some studies have identified the involvement of phospholipase D in PC metabolism from gut microbiota, indicating that intestinal microorganisms are potential targets for phospholipid metabolism and TMA inhibition (Chittim et al., 2019; Orman et al., 2019). Herein, drug-responsive microbial metabolites in Tg mice after rhubarb intervention for 30 and 60 days with consistent trends were further screened by metabolomic analysis. Our study showed that PC (15:0/18:2(9Z,12Z)) was broadly correlated with gut microbiota. Rhubarb reversed the abnormal increase of PC (15:0/18:2(9Z,12Z)) in Tg mice, which suggested that choline metabolism disorders in gut microbiota were alleviated. Patients with inflammatory bowel disease (IBD) harbour a variety of clinically pathogenic bacteria that damaged the integrity of the intestinal barrier by reducing the level of intestinal lysophosphatide, leading to the destruction of the intestinal epithelial barrier and immune activation (Zou et al., 2020). Our results showed that the lysophospholipid LysoPE (14:0/0:0) was associated with *Escherichia-Shigella*, *Marvinbryantia,* and IL-1β. Rhubarb significantly increased the LysoPE (14:0/0:0) level and decreased *Marvinbryantia* abundance in Tg mice. This suggested that rhubarb could affect the metabolism of lysophosphatidylcholine by regulating gut microbiota, thereby playing an important role in reducing inflammation, improving the intestinal barrier. 3-hydroxyundecanoyl carnitine belongs to the acyl carnitine family and structurally contains *O*-acyl carnitine. Our study showed that rhubarb reduced 3-hydroxyundecanoyl carnitine, which was positively correlated with IL-1β, IL-18, Aβ, and *Erysipelatoclostridium*, but negatively correlated with *Bacteroides*. A multi-omic study in the human microbiome program found that the imbalance of acyl carnitine compounds in the feces of patients with IBD with microbiome transcriptome and serum antibodies was accompanied by the reduction of obligatory anaerobes and the overgrowth of facultative anaerobes (Integrative, 2019). These results suggest that 3-hydroxyundecanoyl carnitine may be a key metabolic marker for rhubarb to improve the flora and indicators of AD. Pyroglutamic acid is a cyclized derivative of lactam formed from free amino cyclization of L-glutamic acid (Ponnusamy et al., 2011). Our results showed that rhubarb could significantly increase the levels of glutamate and pyroglutamate in the feces of Tg mice, regulate the metabolism of neurotransmitters related to intestinal microbiota positively, and thus increase the content of neurotransmitters in the brain.

As a biomarker for oxidative stress (Ipson and Fisher, 2016), *o-*tyr is affected by a variety of microorganisms and can bind abnormally to phenylalanine tRNA in cells, producing oxidative proteins that lead to intracellular protein degradation (Bertin et al., 2007; Klipcan et al., 2009), further reducing cellular activity and inhibiting cellular proliferation (Aronson and Wermus, 1965). In this study, we found that *o-*tyr, which continued to increase in the progression of AD, was significantly negatively correlated with a number of neurotransmitters and positively correlated with IL-18. In further *in vitro* experiments, *o-*tyr was proved to increase ROS and NO in neuronal and BV-2 cells as well as to inhibit cellular activity. These results suggested that gut microbiota-derived *o-*tyr might contribute to oxidative stress and neuroinflammation, exacerbating pathological damage in AD.

As the largest endocrine organ in the body, the gut microbial system can produce a wide range of small biologically active metabolites that enter the circulation and affect the brain (Vogt et al., 2017). As AD is a progressive neurodegenerative disease, the gut microbiota and their metabolites accumulate over time as the disease progresses and may exacerbate the process. However, the identified gut microbiota by 16S rRNA sequencing can be highly variable across studies, even yielding completely opposite results regarding a single genus in different studies with the same disease model [6]. Thus, there would be many false positives in screened microbial metabolites by 16S rRNA sequencing-based co-network analysis and presents difficulties for the development of bacteria-targeted drugs for AD. In this study, an improved co-network analysis consisting of RT-PCR and different metabolites combined with pathological indicators provided a more accurate view to uncover the therapeutic mechanisms of rhubarb for AD from the point of gut microbiota. At the very beginning, we explored the microbiota and metabolites that changed with age and correlated with disease indicators. Four bacterial genera were discovered as the drug-responsive bacteria in the process of rhubarb treatment for AD, which were validated using RT-PCR. Then, based on the above identification results, the accuracy of identification for microbial metabolites associated with drug-responsive bacteria was immensely improved by co-network analysis. After that, *o*-tyr was identified and validated its role in promotion of AD pathology by gut-brain transmission. Finally, it demonstrated that rhubarb ameliorated cognitive impairment in Tg mice through decreasing the abundance of *o*-tyr in the gut owing to the regulation of rhubarb-responsive bacteria.

**FIGURE 9:**
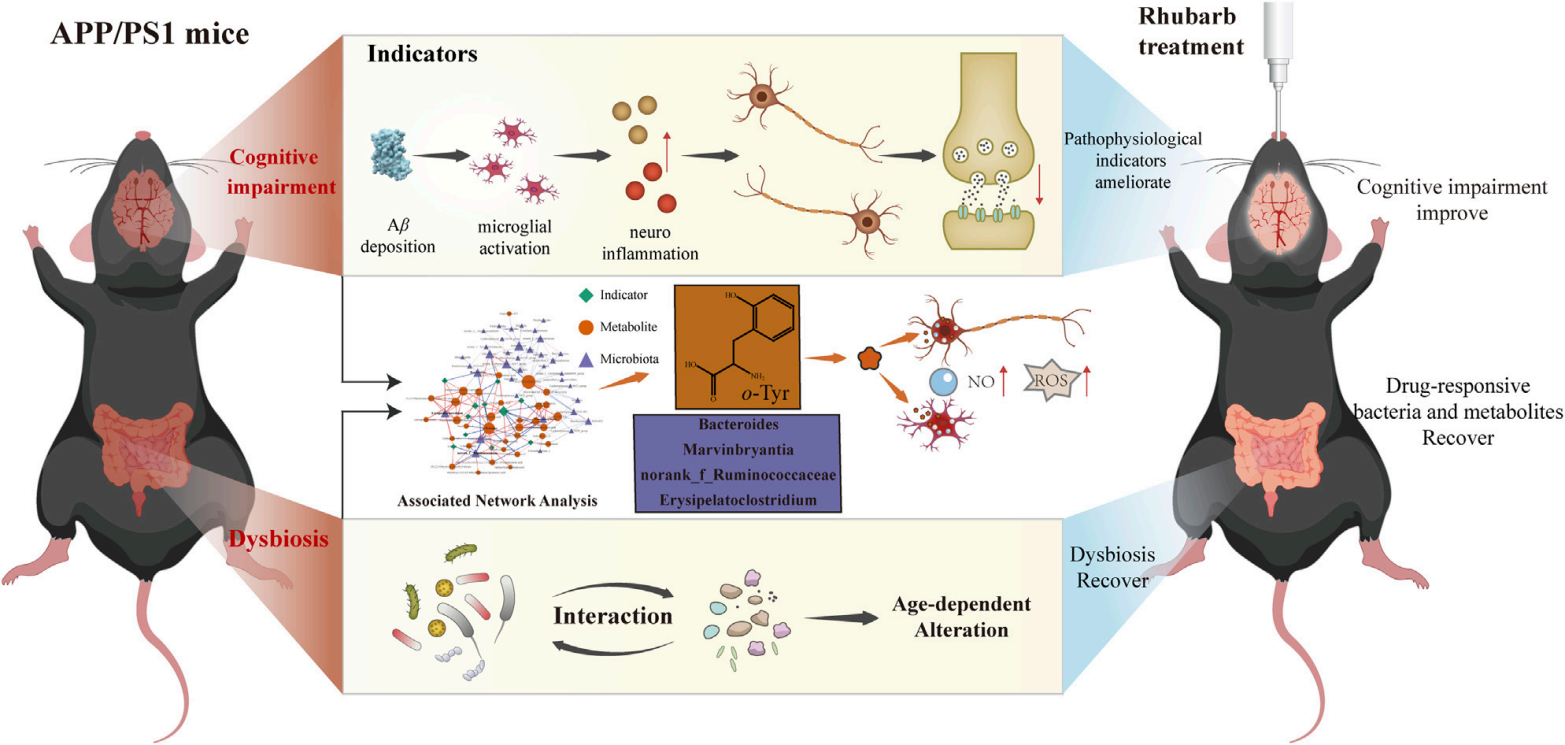
Rhubarb alleviates cognitive impairment in APP/PS1 mice by regulating drug-responsive bacteria and their corresponding microbial metabolites.

In conclusion (Figure 9), our results demonstrated that with the progression of AD, dynamic changes occur in gut microbiota and their corresponding metabolites. Most importantly, a more accurate co-network analysis employed here demonstrated that the therapeutic effects of rhubarb for AD relies on certain bacteria and metabolites correlated with pathological indicators. Reducing the accumulation of metabolites produced by microbiota *in vivo* would help to intervene the progression of AD. The current findings provide a novel perspective on the accurate identification of drug-responsive gut microbes and metabolites to elaborate therapeutic mechanisms of bacteria-targeted drugs for AD.

## Data Availability

The original contributions presented in the study are publicly available. This data can be found here: https://www.ncbi.nlm.nih.gov/bioproject/, PRJNA779704. 210917.

## Glossary

5-HT: serotonin
Aβ: amyloid-β
Ach: acetylcholine
AD: Alzheimer’s disease
ANOVA: the analysis of variance
BBB: blood brain barrier
BPIs: Representative base peak ion chromatograms
CV: coefficient of variation
DA: dopamine
DAF-FM DA: 3-amino,4-aminomethyl-2′,7′-difluorofluorescein diacetate
DCFH-DA: 2′,7′-dichlorodihydrofluorescein diacetate
DMEM: Dulbecco’s modified Eagle’s medium
DMSO: dimethyl sulfoxide
dNTP: deoxynucleoside triphosphate
ELISA: enzyme-linked immunosorbent assay
FBS: fetal bovine serum
FDR: false discovery rate
GABA: γ-aminobutyric acid
Glu: glutamate
HE: hematoxylin and eosin
IBD: inflammatory bowel disease
IL: interleukin
LC-MS: Liquid chromatography–mass spectrometry
LPS: lipopolysaccharides
MRM: multiple reaction monitoring
MS: mass spectrometry
NE: norepinephrine
NO: Nitric oxide
***o*** **-tyr**: *o*-tyrosine
OTUs: operational taxonomic units
PBS: phosphate-buffered saline
PC: phosphatidylcholine
PCA: principal component analysis
PLS-DA: partial least-squares discriminant analysis
QC: quality control
RT-PCR: real-time polymerase chain reaction
ROS: reactive oxygen species
SD: Sprague-Dawley
TCM: Traditional Chinese medicine
Tg: transgenic
TgP: positive drug group
TgR: rhubarb administration group
TMA: trimethylamine
TNF: tumour necrosis factor
UPLC: ultra-performance liquid chromatography
UPLC-Q-TOF/MS: Ultra-Performance Liquid Chromatography-quadrupole time-of-flight mass spectrometry
UPLC-TQ/MS: Ultra-Performance Liquid Chromatography-Triple Quadruple Mass Spectrometry
VIP: variable importance of projection
WT: wild type control group
