# Supplementary material for: Rheum tanguticum Alleviates Cognitive Impairment in APP/PS1 Mice by Regulating Drug-Responsive Bacteria and Their Corresponding Microbial Metabolites
Source: Front Pharmacol. 2021 Dec 15;12:766120. doi: 10.3389/fphar.2021.766120 (PMC8715007; doi:10.3389/fphar.2021.766120)
Supplement: Supplementary file 1 [file DataSheet1.docx]

Supplementary Material

## Supplementary Figures


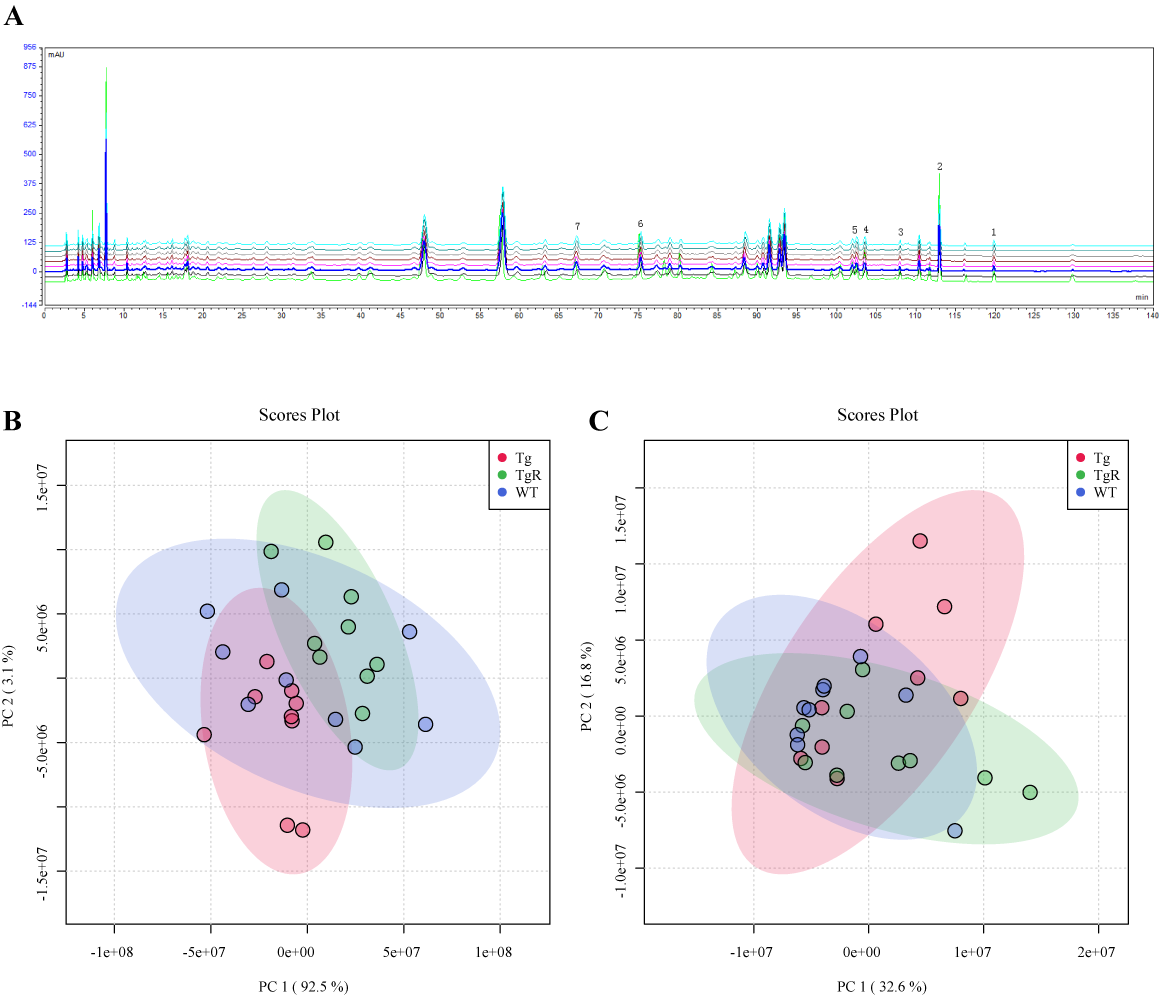


**Supplementary Figure 1.** The influence of rhubarb extraction on fecal metabolic profiles. **A** The fingerprint for the rhubarb decoction. The fecal metabolic profiles of WT, Tg, and TgR mice after 30 days (**B**) and 60 days (**C**) of rhubarb intervention under the positive ion mode.

**
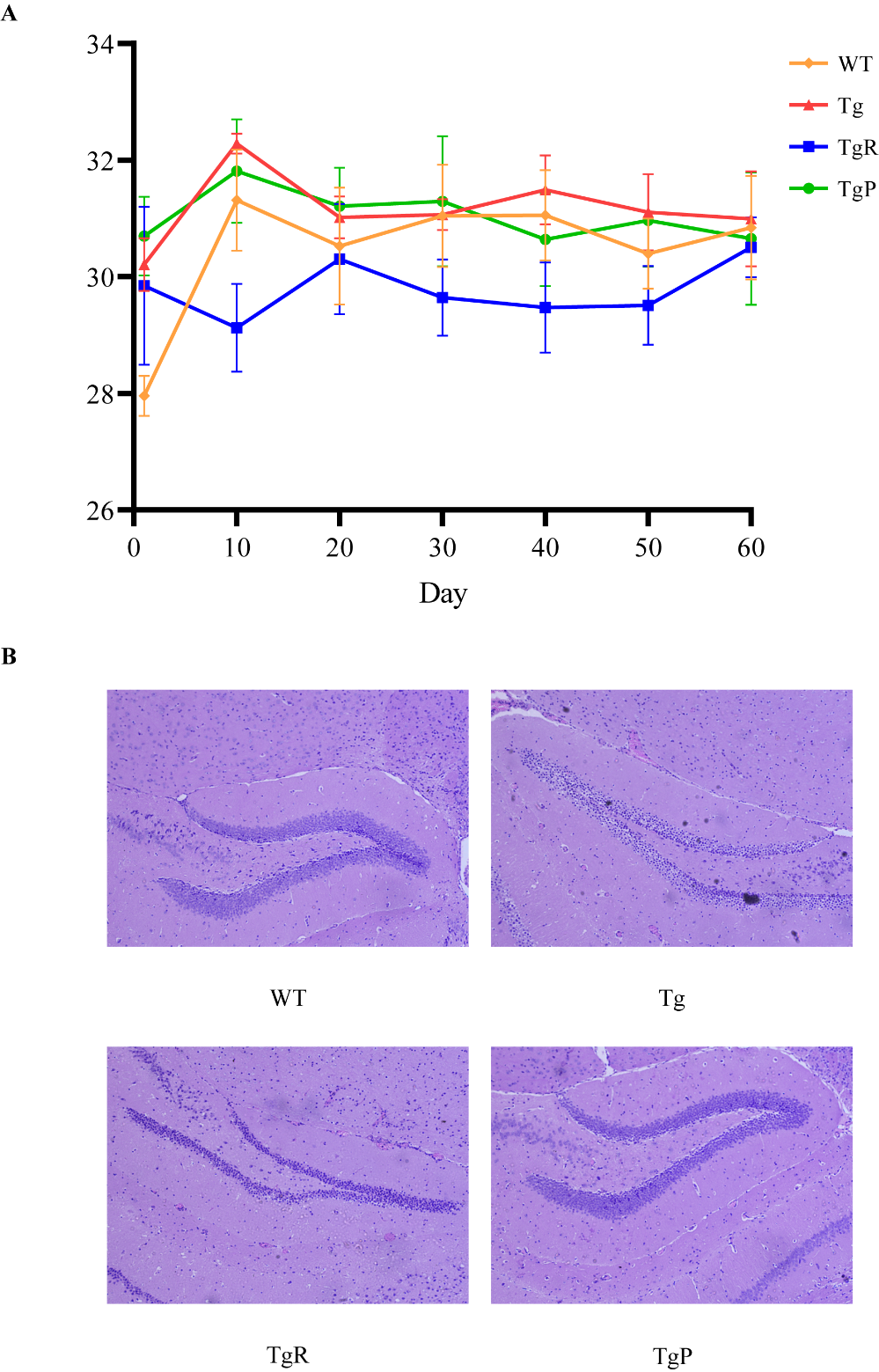
**

**Supplementary Figure 2.** The general health condition of all mice during the experiment. **A** The body weight changes of all mice. **B** Brain tissues of all mice by H&E staining.


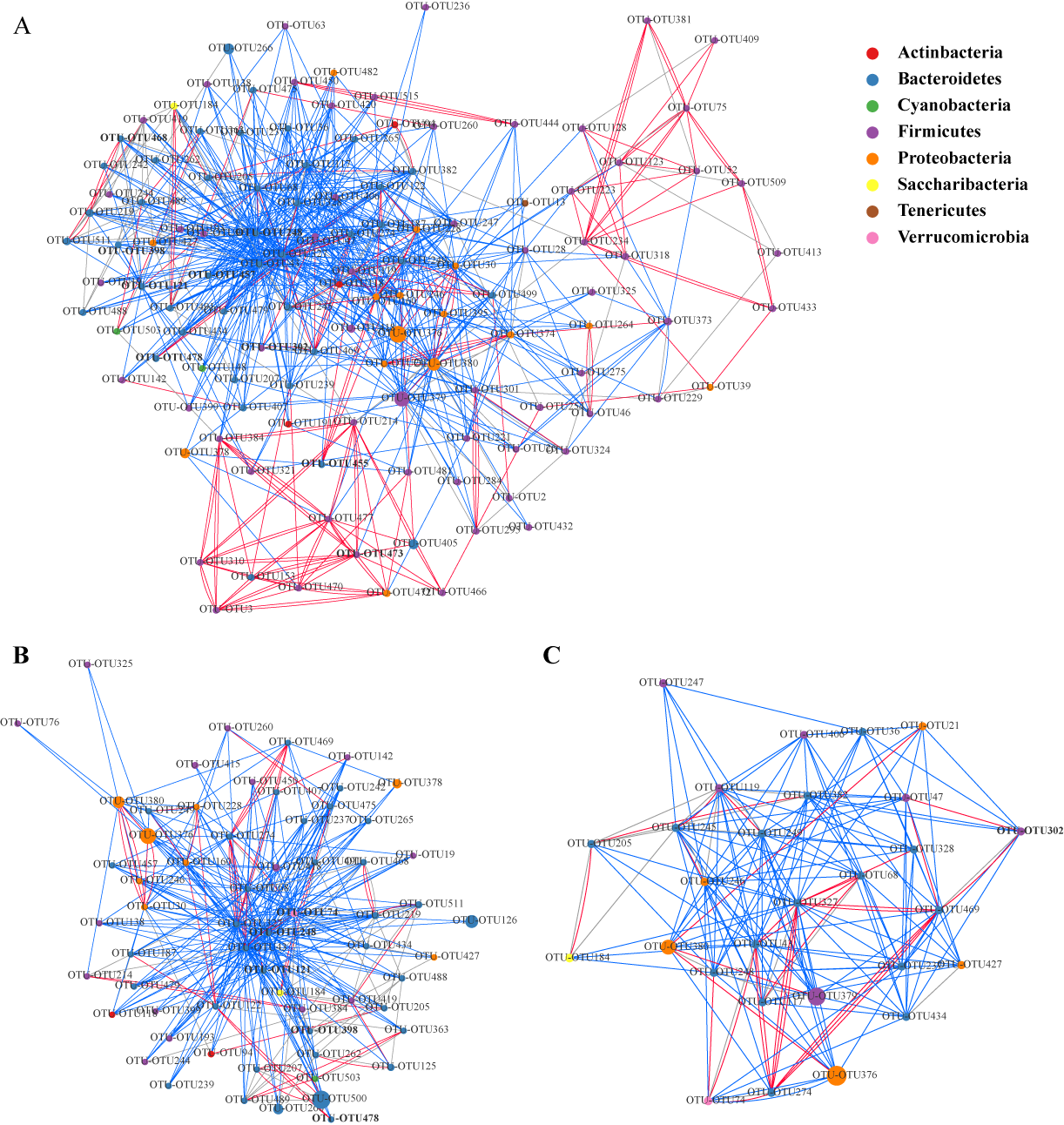


**Supplementary Figure 3.** Subnet analysis of the Co-Net network based on clustering results (**A** complex 1; **B** complex 2; **C** complex 3). The size of the node indicates the relative OTU abundance; the color of the node represents different phyla; the lines between nodes indicate co-abundance (red) or co-exclusion (blue) between the two nodes. OTUs belonging to genera that share a common and consistent trend in all Tg groups are shown in bold font.


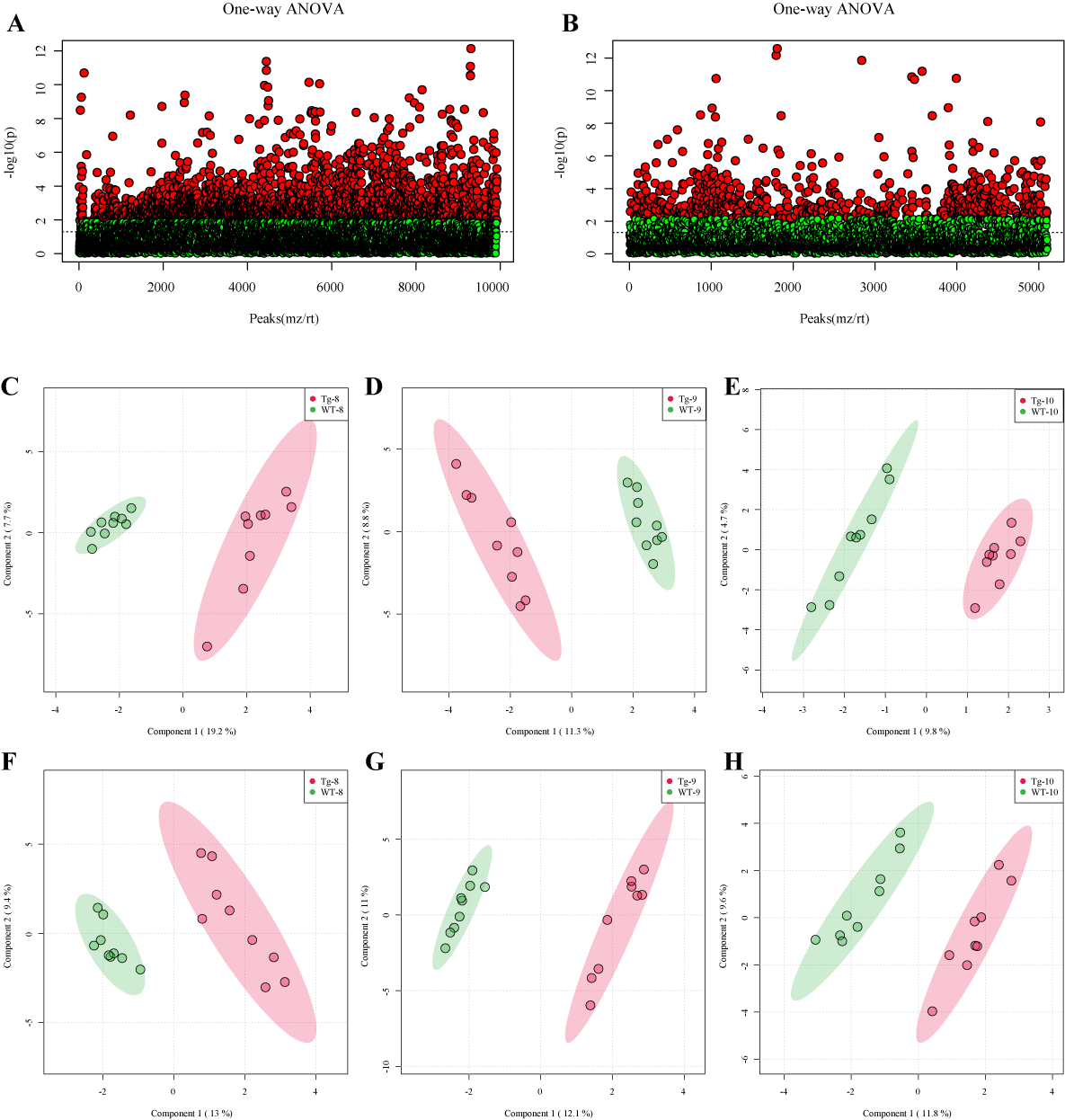


**Supplementary Figure 4.** Screening of differential metabolites between WT and Tg groups. The metabolites among groups with p < 0.05 were screened using the ANOVA t-test (**A**, positive ion mode; **B**, negative ion mode). The PLS-DA score plot obtained from WT and Tg groups of the same age in positive and negative ion modes, **C** and **F** 8 months; **D** and **G** 9 months; **E** and **H** 10 months


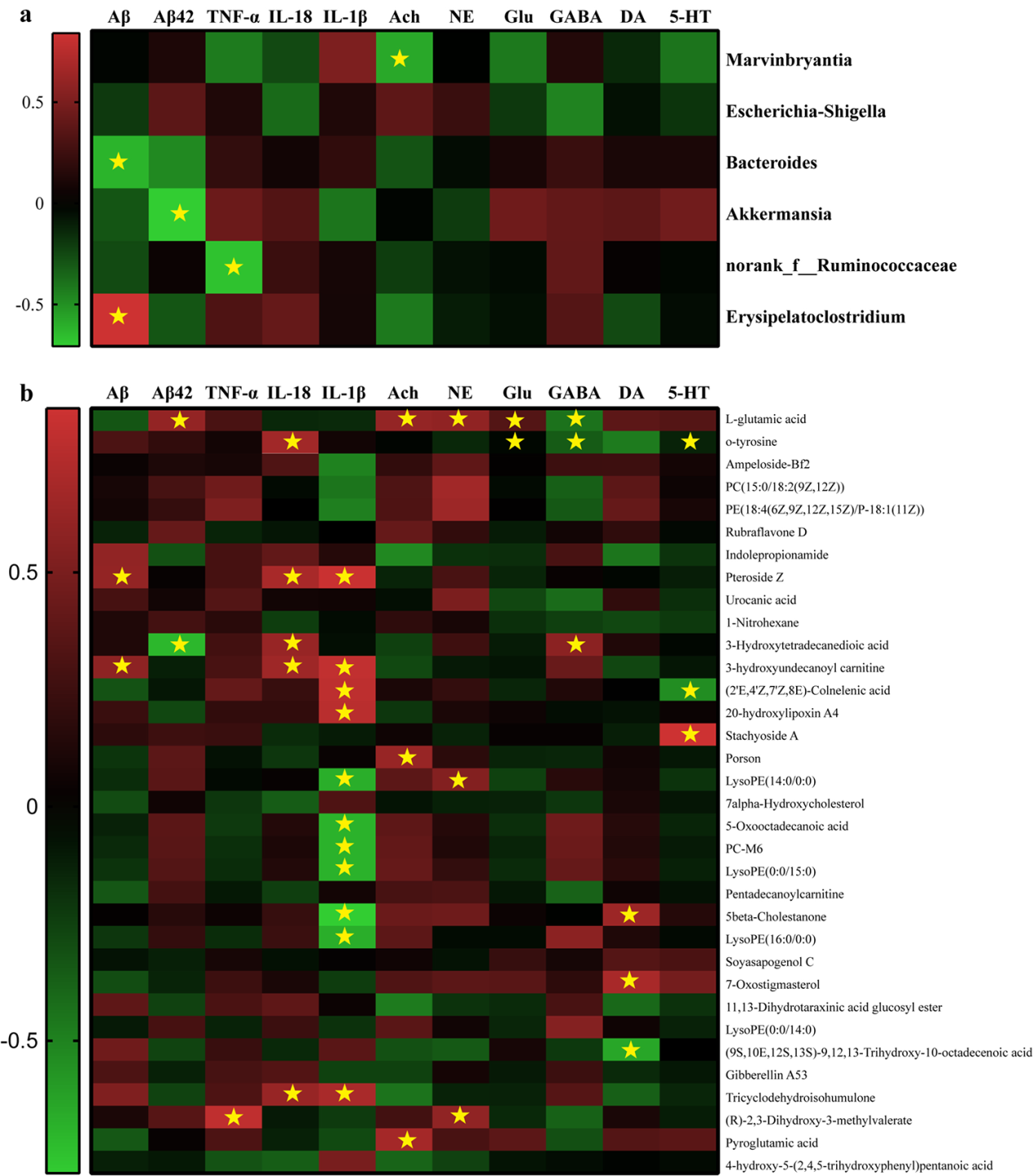


**Supplementary Figure 5.** Pearson’s correlations between pathophysiological indicators, microbiota, and metabolites in the indicators-microbiota-metabolite network. **A**, The heatmap of the correlations between indicators and microbiota; **B**, The heatmap of the correlations between indicators and metabolites. Squares in red (positive correlation) or green (negative correlation) with a yellow star (★) indicate significant correlations with *p*-values < 0.05.

# Supplementary Materials and Methods

## Behavioral test

### Morris water maze test

The Morris water maze test was performed as described by Vorhees and Williams (Vorhees and Williams, 2006). The test was hypothetically divided into four quadrants. The third quadrant contained a platform with a diameter of 6 cm hidden 1.0 cm under the water surface. During the trial, curtains with unique geometric figures were placed at all sides of the pool to avoid visual interference. The spatial learning phase consisted of four trials per day for 6 days, with one additional day (day 7) for a probe trial. During 6 consecutive days of continuous training, each mouse was trained to locate the hidden platform from the starting point with a deadline of 60 s. If the mouse did not find the platform within 60 s, it was allowed to stay on the platform for 10 s. On the 7th day, the platform was removed from the tank and the probe trial was performed. The mouse was allowed to swim freely for 60 s and the number of platform crossings and the time spent in the target quadrant were recorded.

### Step-down test

The test was conducted with an aluminum and acrylic box measuring 200 mm × 75 mm, a floor consisting of a grid of steel bars with a spacing of 12.5 mm (Insight EP 104R), and an adapted platform with a height of 2 cm in the center of the grid. In the training phase, each animal was gently placed on the platform. When the animal stepped down from the platform onto the grid with four paws, it received a 0.7 mA foot shock (stimulus) for 2 s. The animal was then removed from the apparatus. After 24 h (test session), each animal was placed on the platform again. In this session, no shock was applied when the animal stepped down onto the grid. After stepping down, the animal was withdrawn from the apparatus and placed back in its home cage. The time the mouse spent on the platform before stepping down on the grid (step-down delay) was measured by a trained researcher during the training and test sessions with a manual chronometer. The step-down delay threshold was 300 s in the training and test sessions, and animals that did not step down within 300 s were excluded from analysis (Ruan et al., 2016).

## 16S rRNA microbial community analysis

### RNA extraction and PCR amplification

Microbial DNA was extracted from the fecal samples of the WT, Tg, and TgR groups using the OMEGA-soil DNA Kit (D5625, Omega Bio-Tek, USA) according to the manufacturer’s protocols. The final DNA concentration and purification were determined with the NanoDrop 2000 UV-vis spectrophotometer (Thermo Scientific, Wilmington, USA) and the DNA quality was checked with 1% agarose gel electrophoresis. The V3-V4 hypervariable regions of the bacteria 16S rRNA gene were amplified with the primers 5’-sampleIDtag-ATTACCGCGGCTGCT-3’ and 5’-sampleIDtag-CCTACGGGAGGCAGCAG-3’ using the thermocycler PCR system (GeneAmp 9700, ABI, USA). The PCR reactions were conducted using the following program: 3 min of denaturation at 95ºC, 27 cycles of 30 s at 95ºC, 30 s for annealing at 55ºC, 45 s for elongation at 72ºC, and a final extension at 72ºC for 10 min. PCR experiments were performed in triplicate with a 20 μL mixture containing 4 μL of 5 × FastPfu Buffer, 2 μL of 2.5 mM deoxynucleoside triphosphate (dNTP), 0.8 μL of each primer (5 μM), 0.4 μL of FastPfu Polymerase, and 10 ng of template DNA. The resulting PCR products were extracted from a 2% agarose gel, further purified using the AxyPrep DNA Gel Extraction Kit (Axygen Biosciences, Union City, CA, USA), and quantified using QuantiFluor™ -ST (Promega, USA) according to the manufacturer’s protocol.

### Illumina MiSeq sequencing

Purified amplicons were pooled in equimolar and paired-end sequences (2 × 300) on an Illumina MiSeq platform (Illumina, San Diego, USA) according to the standard protocols provided by Majorbio Bio-Pharm Technology Co. Ltd. (Shanghai, China).

### Processing of sequencing data

Raw fastq files were quality-filtered using Trimmomatic and merged with FLASH using the following criteria: (i) The reads were truncated at any site with an average quality score < 20 over a 50 bp sliding window. (ii) Sequences with overlaps longer than 10 bp were merged according to the overlaps with mismatches of no more than 2 bp. (iii) The sequences of each sample were separated based on barcodes (exact matching) and primers (allowing two-nucleotide mismatching) and reads containing ambiguous bases were removed.

Operational taxonomic units (OTUs) were clustered with a 97% similarity cutoff using UPAR (version 7.1 http://drive5.com/uparse/) with a novel ‘greedy’ algorithm that performed chimera filtering and OTU clustering simultaneously. The taxonomy of each 16S rRNA gene sequence was analyzed with the RDP Classifier algorithm (http://rdp.cme.msu.edu/) against the SILVA (SSU123) 16S rRNA database using a confidence threshold of 70%.

### Data analysis

Association network analysis was performed using the Co-Net v1.1.1. beta tool (Faust and Raes, 2016) on Cytoscape v3.7.2. (Shannon et al., 2003). Taxa below a sum of 120 and 12 occurrences per condition were discarded and the relative abundances were calculated. Networks were inferred based on the 1000 top and bottom edges for each of the Pearson, Spearman, Bray, and Kullback-Leibler correlation methods with 1000 iterations. The final *p*-values were computed during bootstrapping and adjusted with Benjamini-Hochberg correction for multiple testing.

## RT-PCR

The total bacterial DNA was extracted from the fecal samples of mice in each group with the E.Z.N.A ™. Stool DNA kit (D4015, Omega Biotek, US), and the procedures were carried out according to the manufacturer’s instructions. First, fecal samples were removed from a -80ºC refrigerator, 200 mg of feces from each sample was measured into a 1.5 mL sterile centrifuge tube, and genomic DNA was detected with 2% agarose gel electrophoresis. For detection of the bacteria *Marvinbryantia* (Desai et al., 2016) and *Erysipelatoclostridium* (Zakham et al., 2019), extracted bacterial DNA was subjected to RT-PCR using the CFX Connect real-time PCR system (Bio-rad Laboratories, Hercules California, US). PCR was performed with a 10 μL sample containing 5 μL SG Green qPCR Mix (with ROX Q1002, SinoGene), 0.2 μL 20 μm upstream and downstream primers, 1 μL bacterial genomic DNA template, and 3.6 μL deionized water. In the blank control, the template DNA was replaced with deionized water. The PCR cycle reaction conditions were as follows: first, pre denaturation was performed at 95ºC for 3 min; the cycle process was denaturation at 95ºC for 10 s, annealing at 55ºC for 20 s, and extending at 72ºC for 10 s, with a total of 40 cycles; at 72ºC, the final extension was 1 min, and the fluorescence signal collection was performed at 72ºC; after the reaction, the melting curve temperature was set at 60-95ºC and increased by 0.5ºC/s. For detection of the bacteria *Bacteroides* (Haugland et al., 2010) and *norank_f_Ruminococcaceae* (Jiang et al., 2017), extracted bacterial DNA was subjected to RT-PCR using the LineGene 9600 Plus real-time PCR system (Bioer Technology, Hangzhou, China). PCR was performed with a 20 μL sample containing 10 μL ChamQ SYBR Color qPCR Master MiX (Vazyme Biotech Co., Ltd, Nanjing China), 0.4 μL 5 μm upstream and downstream primers, 2 μL bacterial genomic DNA template, and 7.2 μL deionized water. In the blank control, the template DNA was replaced with deionized water. The PCR cycle reaction conditions were as follows: first, pre denaturation was performed at 95ºC for 3 min; the cycle process was denaturation at 95ºC for 30 s, annealing at 60ºC for 30 s, extension for 40 s at 72ºC for a total of 35 cycles, and fluorescence signal acquisition at 72ºC. After the reaction, the melting curve temperature was set at 60-95ºC and increased by 0.5ºC/s. **Table 1** shows the sequences for each primer set, which targeted the 16S rRNA genes for each bacteria group. Each standard curve was prepared based on the cell numbers measured using a bacterial counting chamber with each strain indicated. DNA from each standard strain was extracted as described above and used for RT-PCR. All RT-PCR experiments were performed with duplicates for each sample.
